# Zebrafish as a model for Catel–Manzke syndrome—identification and characterization of the zebrafish *TGDS* ortholog

**DOI:** 10.1111/febs.70307

**Published:** 2025-10-29

**Authors:** Maria Rosaria Coppola, Deianira Bellitto, Erfan Asgari, Virginia Bazzurro, Giorgia Casucci, Francesco Piacente, Matteo Bozzo, Davide Ceresa, Costantino Parisi, Cecilia Winata, Simona Candiani, Michela Tonetti

**Affiliations:** ^1^ Department of Experimental Medicine University of Genova Italy; ^2^ Department of Earth, Environment and Life Sciences University of Genova Italy; ^3^ IRCCS Policlinico San Martino Genoa Italy; ^4^ International Institute for Cell and Molecular Biology Warsaw Poland; ^5^ Present address: Department of Pharmacy and Biotechnology University of Bologna Italy; ^6^ Present address: IRCCS Policlinico San Martino Genova Italy

**Keywords:** Catel–Manzke syndrome, craniofacial development, CRISPR/Cas9, *TGDS*, zebrafish

## Abstract

Catel–Manzke syndrome (CMS) is a rare genetic disorder associated with mutations in the TDP‐glucose 4,6‐dehydratase (*TGDS*) gene, the function of which in vertebrates remains unclear. This study investigated the zebrafish ortholog *tgds* to assess its suitability for modeling the disease. During development, the *tgds* transcript exhibits a conserved biphasic expression pattern with an initial maternal contribution followed by a second wave of expression after gastrulation. Recombinant zebrafish *Tgds* expressed in *Escherichia coli* demonstrated UDP‐D‐glucose 4,6‐dehydratase (EC4.2.1.76) activity, similar to *TGDS* orthologs in lower eukaryotes, where it acts as the first step in the L‐rhamnose biosynthetic pathway. This finding suggests the presence of a yet unidentified pathway in vertebrates. Furthermore, CMS‐associated mutations in conserved residues significantly impair enzyme activity and stability. CRISPR/Cas9‐mediated F_0_ knockout of *tgds* resulted in a range of developmental defects in zebrafish. In particular, craniofacial cartilage alterations, associated with a decrease in sulfate glycosaminoglycan content, mirrored some skeletal features observed in humans with CMS. These findings establish the zebrafish as a relevant model to further explore CMS pathogenesis and the *in vivo* function of *tgds*.

## Graphical abstract

Zebrafish Tgds, when expressed as a recombinant protein, catalyzes the dehydration of UDP‐D‐glucose, the initial step in the formation of 6‐deoxyhexoses. Corresponding Tgds mutations found in Catel–Manzke syndrome patients lead to reduced enzymatic activity and stability. Furthermore, F_0_ knockout zebrafish larvae exhibit craniofacial cartilage malformations, reminiscent of the human syndrome, along with reduced levels of sulfated glycosaminoglycans (sGAG).

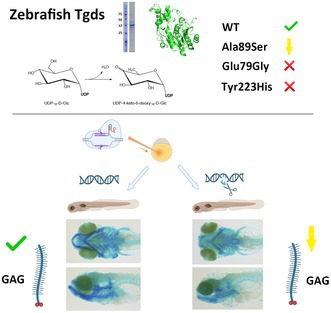

**Abbreviations**
CMS: Catel–Manzke syndrome
D‐Glc: D‐glucose
D‐GlcA: D‐glucuronic acid
dpf: days postfertilization
D‐Xyl: D‐xylose
GAG: glycosaminoglycans
hpf: hours postfertilization
L‐Rha: L‐rhamnose
RNP: ribonucleoprotein
sgRNA: synthetic guide RNA
UDP‐KDG: UDP‐4‐keto‐6‐deoxyglucose
ZGA: zygote genome activation

## Introduction

Catel–Manzke syndrome (CMS) (OMIM # 616145) is a rare autosomal recessive disorder characterized by the Pierre Robin sequence (micrognathia, glossoptosis, and cleft palate) and clinodactyly of the index fingers due to an extra phalanx [1, 2, 3]. Additional skeletal abnormalities include a short neck, talipes, knee dislocation, and joint laxity. Cardiovascular defects, particularly septal and aortic malformations, and short stature are also frequently observed. Atypical CMS cases lacking the full Pierre Robin sequence and clinodactyly have been reported [4, 5, 6]. Affected individuals carry homozygous or compound heterozygous mutations in the *TGDS* gene [1, 6].

Differential diagnoses include Temptamy preaxial brachydactyly syndrome (caused by *CHSY1* mutations [7]) and Desbuquois dysplasia type 1 (caused by *CANT1* mutations [8]). Notably, mutations in *IMPAD1* (encoding 3′(2′), 5′‐bisphosphate nucleotidase 2) were found in two patients initially classified as CMS‐like, leading to chondrodysplasia with joint dislocations, Gpapp type [9]. All these genes encode ER/Golgi‐resident enzymes involved directly or indirectly in glycosaminoglycan (GAG) formation. Phylogenetic analyses have identified *TGDS* as a paralog of *UXS1*, an ER/Golgi‐resident enzyme responsible for UDP‐D‐xylose (D‐Xyl) production from UDP‐D‐glucuronic acid (D‐GlcA) [10]. This finding, coupled with the clinical similarities between CMS and diseases affecting GAG formation, led to the suggestion of a potential role for *TGDS* in D‐Xyl formation, the initial sugar linking chondroitin and heparan sulfate to the proteoglycan core protein [11]. However, direct biochemical evidence supporting the involvement of *TGDS* in GAG metabolism is lacking. Interestingly, biallelic mutations in *KYNU*, encoding an enzyme in the NAD(P)^+^ synthesis pathway, were identified in three previously diagnosed CMS patients, although the link to their clinical manifestations remains unclear [11, 12]. A phenotype resembling CMS was observed in *Tgds*‐mutant mice generated by ethylnitrosourea (ENU) mutagenesis, which exhibited severe micrognathia and cleft palate reminiscent of the Pierre Robin sequence [13].

**Fig. 1:**
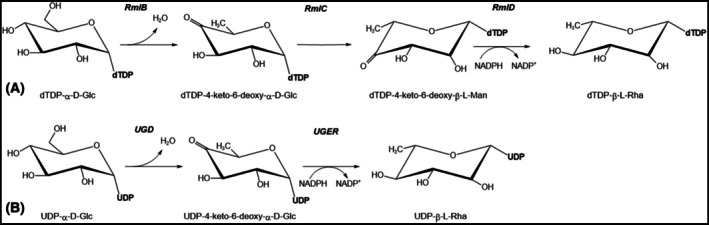
The L‐rhamnose biosynthetic pathway in prokaryotes and eukaryotes. (A) In Bacteria and Archaea, the biosynthesis of TDP‐β‐L‐rhamnose begins with the enzyme RmlB, which catalyzes the dehydration of TDP‐α‐D‐glucose, resulting in the formation of an unstable intermediate, TDP‐4‐keto‐6‐deoxy‐α‐D‐glucose. This intermediate then undergoes two subsequent enzymatic steps: a 3,5‐epimerization catalyzed by RmlC, and a stereospecific NADPH‐dependent 4‐keto‐reduction catalyzed by RmlD, ultimately yielding TDP‐β‐L‐rhamnose. Notably, Nematoda utilize a similar pathway, also employing the TDP‐bound substrate. (B) In contrast to the bacterial and archaeal pathway, most eukaryotes and giant viruses employ a slightly different strategy for L‐rhamnose biosynthesis. Here, the initial dehydration reaction, catalyzed by UGD (UDP‐D‐glucose 4,6‐dehydratase, a homolog of RmlB), utilizes UDP‐α‐D‐glucose as the substrate. The subsequent epimerization and reduction steps are typically carried out by a single, bifunctional enzyme resembling RmlD, termed UGER (UDP‐4‐keto‐6‐deoxy‐D‐glucose epimerase/reductase). Interestingly, higher plants exhibit further evolutionary adaptation, where the RmlB‐like and RmlD‐like domains are fused into single polypeptide chains known as RHM proteins.

The human *TGDS* gene is currently annotated as TDP‐D‐glucose (D‐Glc) 4,6‐dehydratase (EC4.2.1.46) based on its homology to bacterial and archaeal RmlB enzymes, which catalyze in the first step of the TDP‐L‐rhamnose (L‐Rha) biosynthetic pathway (Fig. 1A) [14] and other modified 6‐deoxyhexoses. Functional orthologs of these bacterial dehydratases exist in plants, giant viruses, lower eukaryotes, and Nematoda [15, 16, 17, 18, 19], all of which possess a well‐characterized L‐Rha pathway. Except for nematodes, all the eukaryotic dehydratases so far characterized utilize UDP‐D‐Glc as a substrate (Fig. 1B). Orthologs with high sequence identity to the UDP‐D‐Glc 4,6‐dehydratases from lower eukaryotes are also present in higher animals [17]. However, endogenous L‐Rha synthesis has never been described in vertebrates, and this sugar is absent from their complex glycans. Consequently, the function of *TGDS* in human cells remains unknown, and its role in CMS pathogenesis is elusive.

Zebrafish have proven valuable for elucidating the biological activity and pathogenic mechanisms of human disease‐related gene orthologs through morphants and transgenic models [20]. Their increasing use stems from several advantages, including high fecundity, external development, rapid life cycle, small size, and larval transparency. Furthermore, early developmental stages are conserved among vertebrates. These characteristics make zebrafish a good alternative to mouse models, particularly for analyzing the impact of gene disruption during early development. CRISPR/Cas9 technology has now provided an efficient tool for generating targeted mutations, overcoming some limitations of morpholino‐based knockdown experiments.

The *tgds* gene was only annotated in the very recently released GRCz12tu reference genome. However, information regarding its expression in adult tissues and during development is still scarce. Therefore, the initial objective was to examine the expression patterns during development and in adult tissues. Importantly, we have also demonstrated that the recombinant zebrafish Tgds protein, expressed in *E. coli*, exhibited an UDP‐D‐Glc 4,6‐dehydratase activity. This represents the first demonstration that a vertebrate Tgds has the same enzymatic activity observed in lower eukaryotes. Our findings also revealed that CMS‐associated mutations significantly impair both the stability and activity of the zebrafish Tgds protein, providing the first biochemical evidence, beyond genetic analyses, for the involvement of TGDS in CMS. Finally, as a proof of principle, we targeted *tgds* via CRISPR/Cas9 F_0_ knockout in zebrafish, which also revealed phenotypes associated with CMS, establishing a foundation for future studies using transgenic zebrafish models to investigate CMS pathogenesis.

## Results

### Sequence analysis of the *tgds* gene and protein isoforms

The *tgds* gene has been very recently annotated in the last zebrafish reference genomes (GRCz12tu), while it was absent or incomplete in the previous versions (GRCz10 and GRCz11). A single copy of the *tgds* gene, comprising 13 exons, is located at the end of the long arm of chromosome 6. Synteny of this genomic region with other vertebrate genomes is limited, with only one gene, *gpr180*, associated with *tgds*. Moreover, in most vertebrates *tgds* gene is formed by 12 exons instead of 13.

Since our previous studies revealed a complex evolutionary history for the *TGDS* gene family [17], we utilized the gene gain/loss tree available in the Ensembl database, using human *TGDS* as a query, with a focus on the Chordata phylum. The tree revealed the absence of *TGDS* orthologs in Tunicata, such as *Ciona intestinalis*, whereas they are found in Cephalochordata (Branchiostoma) and all Vertebrata, except in Agnatha, which includes the jawless fishes like lamprey and hagfish. The lack of the *tgds* gene in jawless fishes was further confirmed by extensive BLASTn analysis in databases. Two or more *tgds* copies, a typical feature of several other genes in the teleost fish lineage, were found in the Salmonidae only.

The *tgds* gene undergoes alternative splicing, producing seven known transcript isoforms. Four of these are predicted to be protein‐coding, while three are considered noncoding. Transcripts NM_001441693.1 (variant 1) and NM_001441694.1 (variant 2) both encode an identical 347‐residue protein (NP_001428622.1 and NP_001428623.1, respectively). These two transcripts differ only by a six‐base insertion in the 5′ untranslated region (5′ UTR) located between exon 1 and exon 2, upstream of the start codon. For simplicity, we use NP_001428622.1 as our reference sequence.

Transcripts NM_001441695.1 (variant 3) and NM_001441696.1 (variant 4) contain a four‐base insertion from alternative splicing between exon 5 and exon 6. This insertion causes a frameshift, leading to a downstream start codon and a predicted protein of only 244 residues (NP_001428624.1). This shortened protein would lack most of the N‐terminal domain essential for NAD^+^ binding, which is critical for the enzymatic activity and structural stability of extended SDR family proteins. Furthermore, the lack of a canonical Kozak sequence (absence of a purine at the −3 and +1 positions) at the proposed start codon suggests that these transcripts are not translated and are therefore also likely noncoding. The three RNA isoforms designated as noncoding (NR_199835.1, NR_199836.1, and NR_199837.1) include a 10‐nucleotide insertion between exons 10 and 11. This leads to a frameshift and a premature stop codon, resulting in a truncated 247‐amino acid protein. Although this protein was once listed as a reference sequence in NCBI and UniProtKB, it has since been removed. Indeed, our data indicated that these transcripts are expressed at very low levels (not shown).

**Fig. 2:**
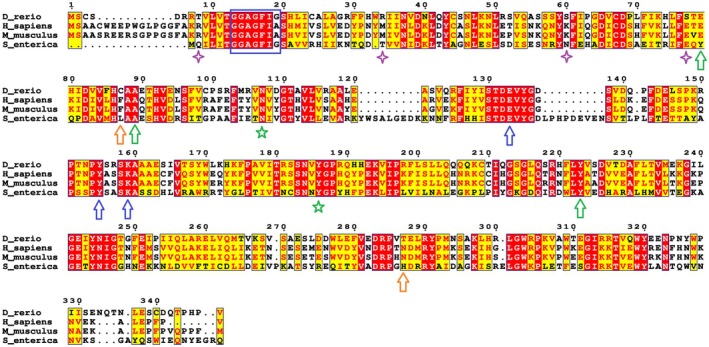
Sequence alignment of zebrafish Tgds with the corresponding orthologs from *S. enterica* and the human TGDS. Red boxes highlight the identical residues, while the yellow boxes refer to conserved ones. The blue box indicates the conserved Gly‐X‐X‐Gly‐X‐X‐Gly/Ala motif characteristic of a Rossmann fold. Residues forming the catalytic triad are marked with blue arrows, while green arrows point to conserved amino acids mutated in CMS and analyzed in this study. Orange arrows indicate the other mutations observed in CMS patients not analyzed in the present study. The green stars and purple diamonds denote the amino acids that establish contacts with the mutated residues and are discussed further in the Results section (‘Properties of the WT and mutant Tgds proteins’). The sequences shown are: *S. enterica* (UniProt Q9EU31, PDB 1G1A), *H. sapiens* (UniProt O95455) and *D. rerio* (GenBank NP_001428622). Protein sequence alignments were performed with Espresso on the T‐coffee server and figures were generated via ESPript.

Figure 2 presents the sequence alignment of the Tgds protein with the human and mouse orthologs and *Salmonella enterica* serovar Typhimurium TDP‐D‐glucose 4,6‐dehydratase (RmlB). The green arrows highlight residues mutated in CMS patients that are conserved in the zebrafish proteins and that were analyzed in this study. Orange arrows highlight the other residues substituted in CMS patients, which are not conserved in the zebrafish protein. The zebrafish and human proteins exhibit approximately 70% sequence identity, with perfect conservation of the typical SDR enzyme Gly‐X‐X‐Gly‐X‐X‐Gly/Ala motif (highlighted by a blue box) and the catalytic triad residues (indicated by blue arrows), suggesting that they are likely functional enzymes. [14, 21]. Zebrafish Tgds identity with the bacterial enzymes is about 37%, while with lower eukaryote proteins is around 41%.

### Expression of *tgds* during development and in adult zebrafish tissues

**Fig. 3:**
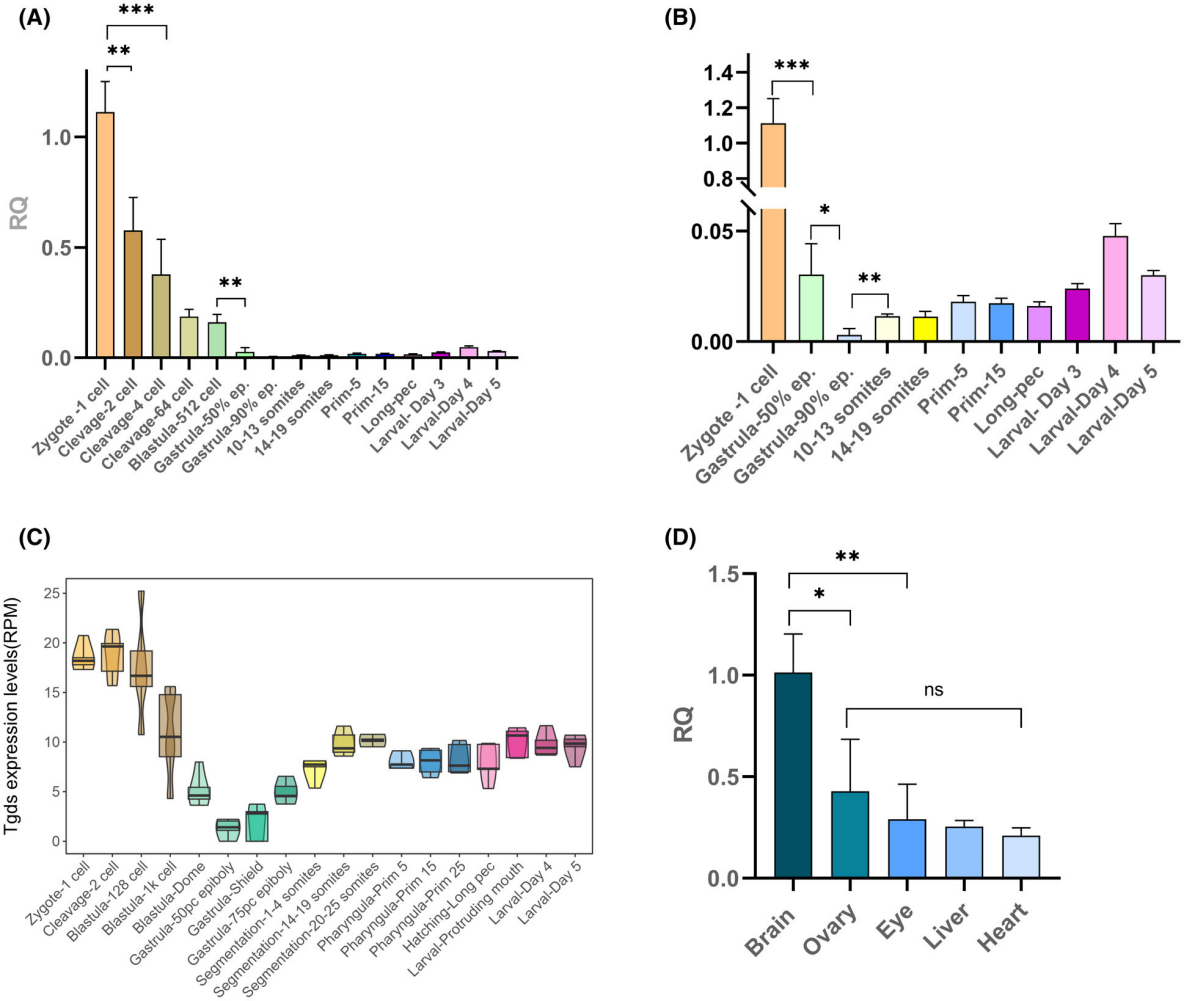
Expression of *tgds* transcripts in adult tissues and during zebrafish development. (A) The expression dynamics of *tgds* were examined during zebrafish development via RT–qPCR. cDNA was generated from samples collected at various developmental stages, and the 1‐cell stage was used as the reference point for normalization. (B) An enlarged view of panel A focuses on *tgds* expression levels in the more advanced stages of development. (C) To complement the targeted expression analysis, publicly available RNA‐seq data from a zebrafish development dataset [22] were analyzed by mapping reads to the *tgds* coding sequence. (D) RT–qPCR was employed to quantify the relative abundance of *tgds* in various adult tissues. The brain tissue served as the reference for normalization in this analysis. Statistical analysis revealed significant differences in *tgds* expression between the brain and other tissues. Welch's *t*‐test was used for the statistical analyses: mean ± SD, obtained from at least three independent replicates for each point is presented (*P < 0.05, **P < 0.01, ***P < 0.005, ns = not significant).

Given that the clinical manifestations of CMS are linked to developmental defects, we analyzed *tgds* expression at different developmental stages. RT–qPCR analyses revealed maternal accumulation of *tgds* in the zygote (Fig. 3A). Following fertilization, a rapid decline in transcript levels is evident during the early cleavage stages, with the lowest expression occurring during gastrulation (between 50% and 90% epiboly). Subsequently, a second wave of transcription, originating from the zygotic genome, starts at the end of gastrulation (Fig. 3B). This biphasic expression pattern, characterized by lower expression during the gastrula stage, is further supported by zebrafish RNA‐Seq data from transcriptome analyses (Fig. 3C) [22]. Intriguingly, transcriptomic data from the XenBase database (https://www.xenbase.org/xenbase/) [23] revealed a similar biphasic expression profile of *tgds* in *Xenopus laevis* and *X. tropicalis* (Fig. S1), with a sharp decline in transcript abundance until the gastrula stage, suggesting that this pattern is conserved in other vertebrates.

Expression analysis on adult tissues revealed higher *tgds* levels in the brain than in the other analyzed tissues; no significant differences were found between the other tissues (Fig. 3D). This expression pattern is also consistent with the results obtained in *X. leavis* (Fig. S1).

**Fig. 4:**
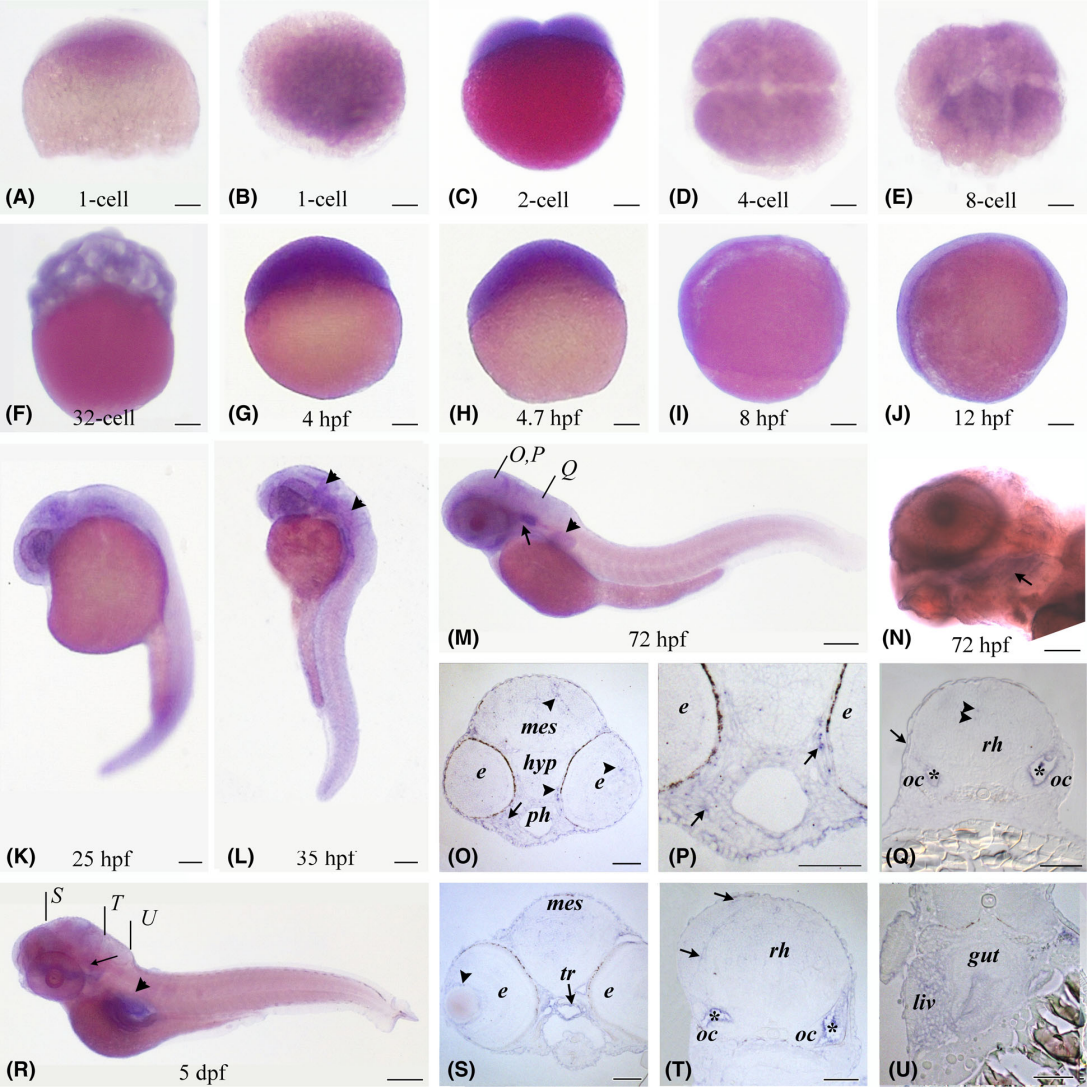
Analysis of *tgds* expression in developing zebrafish by *in situ* hybridization. (A–J) Early stages showing *tgds* expression in the whole embryo. A and B, 1‐cell side and animal dorsal views, respectively. (D, E) Embryos in animal view. (C, F–J) Side views of embryos. (K–M) Late embryonic stages show diffuse expression in the mesencephalic and rhombencephalic regions (arrowheads in L) as well as in the pectoral fin (arrowhead in M) and otic capsule (arrow in M). From 72 hpf, transcripts are also found in the branchial region (arrows in N and R). (N) Enlarged view of the head showing *tgds* expression in the region of the branchial arches (arrow). (O–Q) Sections at the levels indicated in panel M, showing *tgds* expression in some cells of the eye and mesencephalon (arrowheads) and in the mesenchyme of the pharyngeal region (arrows in O and P). More posteriorly (Q), *tgds* is expressed in the otic capsules (asterisks), scattered cells of the rhombencephalon (arrowheads), and ectoderm (arrow). (R) Early larval stage (5 dpf). (S–U) Sections at the levels indicated in panel R. In R, *tgds* expression is visible in the trabecular cartilage (arrow) and in the region of the gut and liver (arrowhead), in the trabecular cartilage (arrow) and the (S) *tgds* is expressed in the lens capsule (arrowhead), in the trabecular cartilage (arrow) and associated epithelium, and in scattered cells of the mesencephalon. (T) *tgds* expression is found in the otic capsules (asterisks) as well as in the cells of the rhombencephalon (arrow). (U) *tgds* transcripts are detected in the gut and liver. Scale bars are 100 μm for whole mounts and 50 μm for sections. e, eye; hyp, hypothalamus; liv, liver; mes, mesencephalon; oc, otic capsule; ph, pharynx; rh, rhombencephalon; tr, trabecular cartilage. Images are representative of three independent ISH experiments.

By whole‐mount *in situ* hybridization (ISH), we confirmed the presence of maternal *tgds* transcripts from the zygote to the onset of gastrulation (Fig. 4A–H). Consistent with our RT–qPCR analyses, we detected weak *tgds* expression during gastrulation, following the maternal‐to‐zygotic transition (Fig. 4G–J). At 25 and 35 hpf, zygotic *tgds* transcripts were primarily concentrated in the anterior part of the embryo, specifically in the developing eyes and brain (Fig. 4K,L). In 72‐hpf embryos, *tgds* was strongly expressed in the optic capsule and mesenchymal cells in the pharyngeal region, as well as in scattered cells in the eye, brain, and epidermis (Fig. 4M–Q). Interestingly, *tgds* is also expressed in the developing pectoral fin (Fig. 4M). At 5 dpf, which is the latest stage analyzed, the expression in the head region was comparable to that in the earlier stages, with *tgds* transcripts found in scattered cells of the brain, the lens capsule of the eye, and the otic capsule (Fig. 4R–T). Notably, *tgds* was still expressed in some cartilaginous derivatives of the pharyngeal mesenchyme, such as the trabecular cartilage (Fig. 4S, arrow). In addition, *tgds* expression was established in the gut and liver (Fig. 4U). A control ISH with the antisense probe is reported in Fig. S2.

### Properties of the WT and mutant tgds proteins

**Fig. 5:**
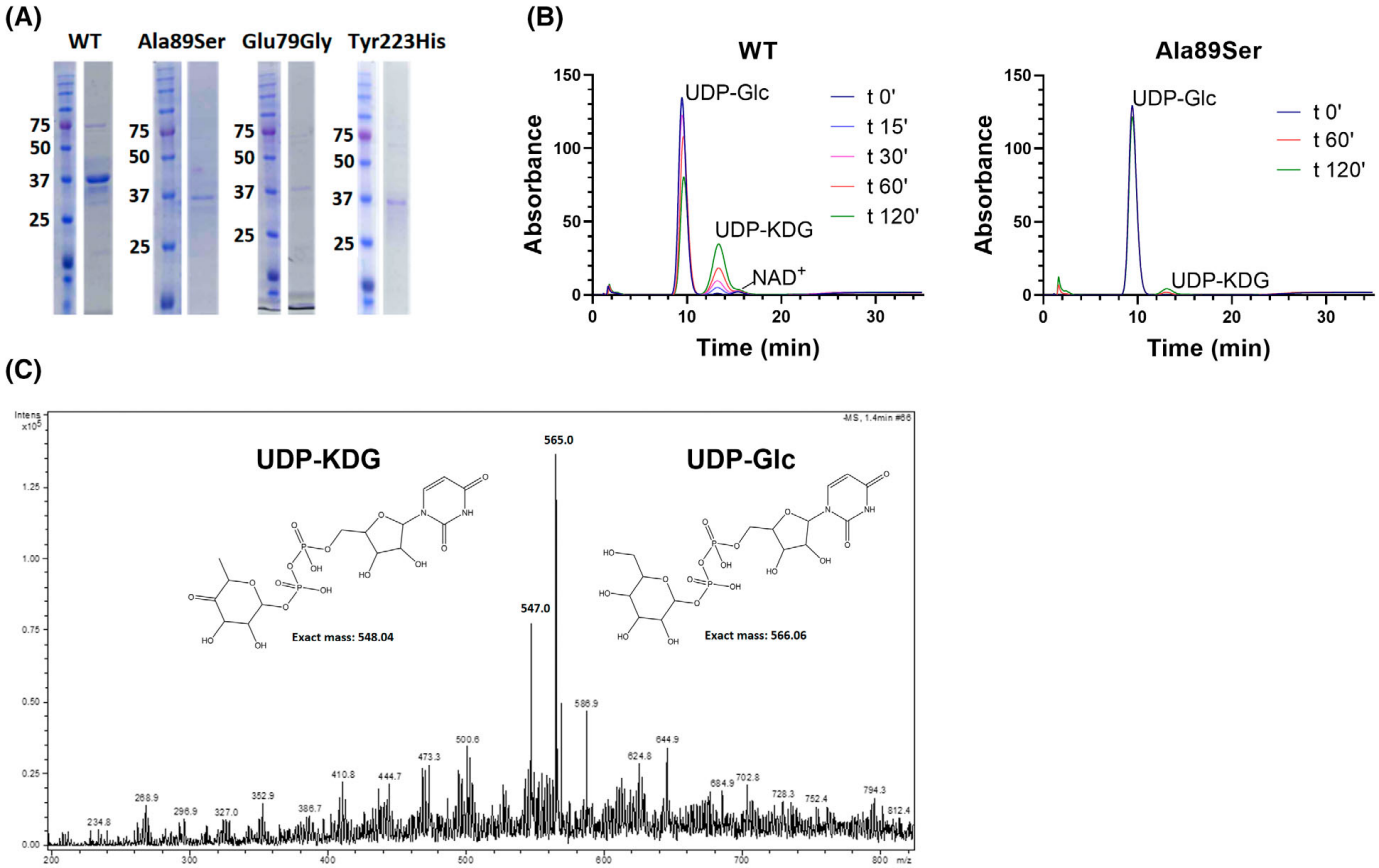
Enzymatic activity and stability of the recombinant WT and mutant Tgds proteins. (A) Representative SDS/PAGE analysis of the WT and mutant Tgds expressed in *E. coli* and purified using a N‐terminal 6XHis tag. (B) Enzymatic activity of recombinant WT and Ala89Ser recombinant proteins, assessed through an *in vitro* assay monitored by anion exchange HPLC. The incubation of WT Tgds with its substrate, UDP‐D‐Glc, resulted in a time‐dependent conversion to the product, UDP‐4‐keto‐6‐deoxy‐D‐Glc (UDP‐KDG), as evidenced by the progressive decrease in the UDP‐D‐Glc peak and the corresponding increase in the UDP‐KDG peak over time. A minor peak corresponding to NAD^+^ is also present, likely due to the release of this tightly bound cofactor from the protein during the denaturation step prior to the HPLC analysis. The retention times of the substrate and product were confirmed using the recombinant *T. vaginalis* UDP‐D‐Glc 4,6 dehydratase [17]. To investigate the role of a specific amino acid residue, the Ala89Ser mutant of Tgds was tested in parallel under identical conditions as the WT enzyme in panel A. HPLC analysis of the reaction with the mutant enzyme is shown, allowing direct comparison of its activity to that of the WT protein. The rate of conversion was determined by measuring the decrease in the UDP‐D‐Glc peak area over the analyzed time range, which exhibited a linear relationship. The data presented in these figures are representative of a single experiment. The cumulative results obtained from three different batches of the recombinant proteins are reported in Table 1. (C) ESI‐MS analysis of UDP‐D‐Glc (*m*/*z* 565.05) tested after incubation with WT Tgds; the peak at 547.04 corresponds to the expected *m*/*z* of the dehydration product, UDP‐KDG. A representative mass spectrum from one of two independent analyses is presented.

Tgds was produced in *E. coli* and purified via an N‐terminal 6xHis tag (Fig. 5A). Enzymatic analysis via anion exchange HPLC demonstrated that Tgds catalyzes the dehydration of UDP‐D‐Glc, yielding UDP‐4‐keto‐6‐deoxy‐D‐glucose (UDP‐KDG), similarly to the results obtained with Tgds orthologs from other organisms already characterized in our laboratory [15, 17] (Fig. 5B). The identity of the product was confirmed by its retention time compared to the control produced by *T. vaginalis* UGD [17] and by ESI‐MS data (Fig. 5C), which were comparable to the ones obtained in our previous studies on UDP‐D‐glucose 4,6‐dehydratases from other organisms [15, 17, 24].

The enzyme exhibited lower activity with TDP‐D‐glucose (approximately 20% of that observed with the UDP‐bound substrate), which is consistent with its eukaryotic origin [15, 17]. Notably, no activity was detected when UDP‐N‐acetyl‐D‐glucosamine, UDP‐D‐galactose, and UDP‐D‐GlcA were tested as substrates. The lack of activity on UDP‐D‐GlcA indicates that Tgds is not directly involved in UDP‐D‐Xyl formation. Consistent with previous findings from our laboratory on *T. vaginalis* and Mimivirus enzymes, the addition of bivalent cations or the NAD^+^ coenzyme, which is required for internal oxidoreduction, did not influence Tgds catalytic activity [15, 17].

Currently, six missense substitutions have been reported in CMS patients: c.298G>T (p.Ala100Ser), c.269A>G (p.Glu90Gly), c.294T>G (p.Phe98Leu), c.700T>C (p.Tyr234His), c.892A>G (p.Asn298Asp), and c.895G>A (p.Asp299Asn) [1, 6]. A frameshift deletion c.270_271del (p.Lys91Asnfs*22) was also reported in one patient. The only substitution observed in homozygosity is the Ala100Ser, assumed to be a founder mutation, while all the others are found in compound heterozygosity. These substitutions are indicated by green or orange arrows in the alignment reported in Fig. 2.

To confirm the detrimental effects of the substitutions observed in CMS patients, we mutated the three perfectly conserved residues between zebrafish and human proteins and expressed them in *E. coli* (Fig. 5A). These mutated residues are highlighted by a green arrow in Fig. 2. Compared with the wild‐type (WT) protein, the Ala89Ser mutant displayed variable but consistently reduced enzymatic activity (Fig. 5B and Table 1). In contrast, Glu79Gly and Tyr223His exhibited nearly undetectable activity and reduced stability (Table 1), suggesting crucial structural roles for these residues. Furthermore, protein recovery after purification of these mutants was generally very low, with the majority of the protein found in inclusion bodies or lost during concentration. ΔΔG^Stability^ calculations via DynaMut2 [25] predicted the Ala89Ser substitution to be neutral/slightly destabilizing, whereas Glu79Gly and Tyr223His were predicted to be medium/highly destabilizing (Table 1), which aligns with the experimental results obtained with the recombinant proteins. The damaging effects of these amino acid substitutions on protein folding were further supported by the lower amounts of tightly bound NAD^+^ (Table 1), a cofactor essential for internal oxidoreduction during the catalytic mechanism [14, 21]. The addition of exogenous NAD^+^ failed to restore catalytic activity or enhance the stability of the mutants.

**Table 1:** Properties of the WT and mutant Tgds proteins.

	Activity (nmol·min^−1^·mg^−1^)^a^	Stability (% loss on day 5)^b^	ΔΔG^Stability^ (kcal·mol^−1^)^c^	NAD^+^/protein molar ratio^d^
WT	1.51 ± 0.20	5 ± 2	–	0.55
Ala89Ser	0.25 ± 0.11	30 ± 6	−0.31	0.39
Glu79Gly	ND^e^	64 ± 16	−1.41	0.16
Tyr223His	ND^e^	50 ± 8	−1.84	0.04

^a^ Activity was determined as indicated in Fig. 5. Data were obtained from three different protein expressions for the WT and mutant strains.

^b^ The stability of the purified WT and mutant Tgds proteins was analyzed at 4 °C. On day 5, the solutions were centrifuged to eliminate the protein precipitates, and the loss of the proteins in the supernatant compared with that on day 1 was determined by measuring the absorbance at 280 nm.

^c^ ΔΔG^Stability^ of the mutants was calculated via DynaMut2.

^d^ Protein molar concentration was determined via a predicted extinction coefficient, as indicated in the text.

^e^ ND, not detected under the experimental conditions used for WT and Ala89Ser mutant analyses.

We then utilized AlphaFold2 models to gain further insight into the effects of these substitutions. The high sequence and structural similarity between the zebrafish and human proteins (Fig. 2 and Fig. S3A), particularly within the active site (the catalytic triad) and the coenzyme‐binding pocket, along with the conservation of the residues mutated in CMS patients, suggest similar functional behavior. We also compared the structure of Tgds to that of the crystallized and well‐characterized *S. enterica* serovar Typhimurium dTDP‐Glc 4,6‐dehydratase (RmlB, 1G1A) [14]; again, very high structural homology was present (Fig. S3B).

**Fig. 6:**
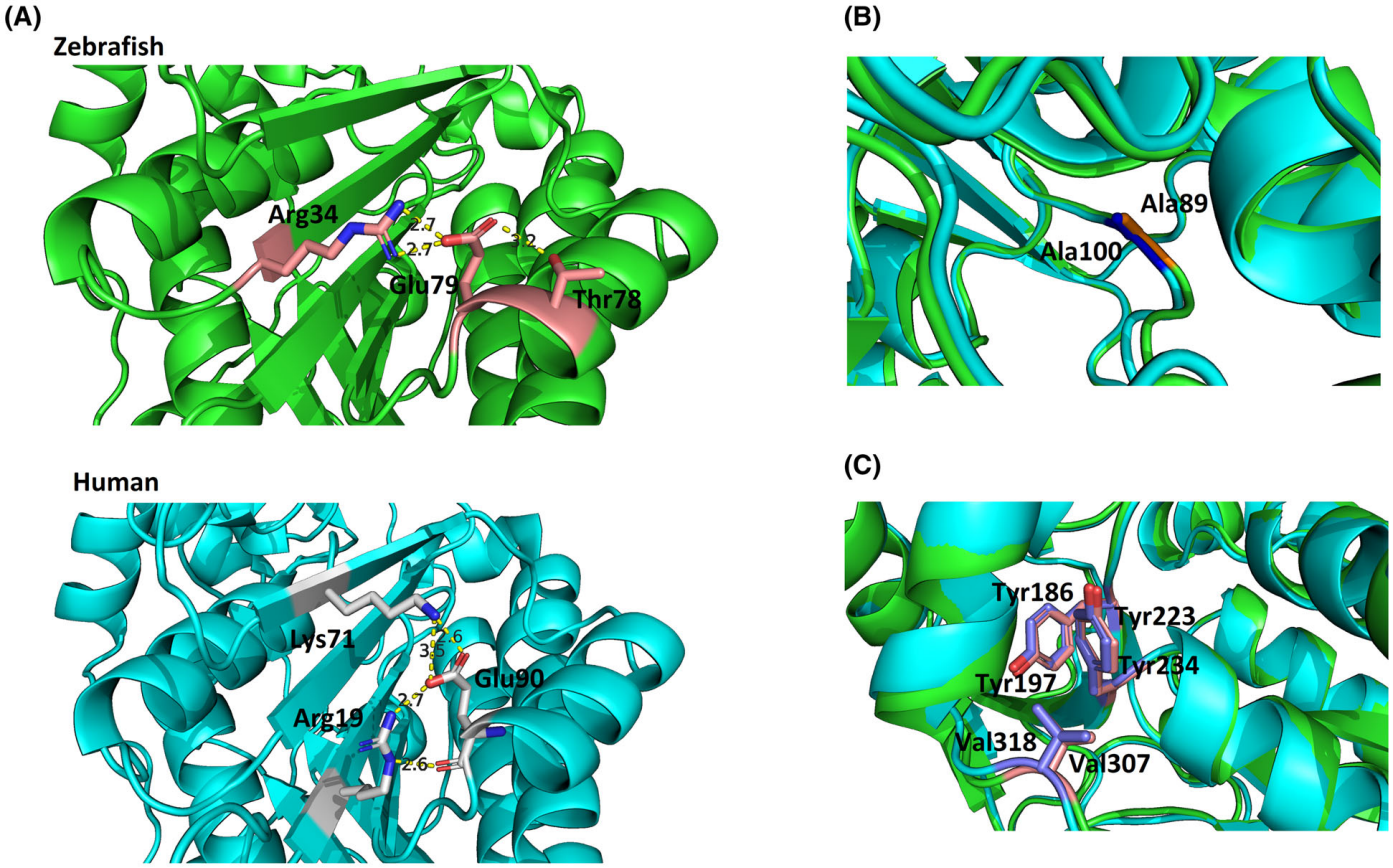
Structural models of zebrafish Tgds and human TGDS, highlighting regions affected by the analyzed amino acid substitutions. Closer views of these regions are shown, with zebrafish Tgds colored green and human TGDS colored cyan. The models were obtained from the AlphaFold database and aligned by PyMOL: zebrafish Tgds: UniProt Q6NYF5, AlphaFold AF‐Q6NYF5‐F1‐v4; human TGDS: UniProt O95455, AlphaFold AF‐O95455‐F1‐v4. (A) In zebrafish Tgds (upper panel), Glu79 forms polar interactions with Arg34 and Thr78. The corresponding residue in human TGDS (lower panel), Glu90, interacts with Arg19 and Lys71. (B) The structural alignment shows perfect superimposition of zebrafish Ala89 and human Ala100. (C) Zebrafish Tyr223 and human Tyr234 are superimposed and exhibit similar interactions with nearby residues.

The Glu79Gly substitution in Tgds could prevent the formation of polar contacts between the lateral chains of Glu79, Arg34, and Thr78. These contacts are supposed to stabilize the α2 helix with the β2 sheet of the Rossmann fold (Fig. 6A, upper panel), thus explaining the low stability observed for the recombinant mutant. In the human TGDS, similar interactions can occur for Glu90, but with Arg19 and Lys71, between the α2 helix and the β1 and β3 strands (Fig. 6A, lower panel), suggesting that mutation of this residue has comparable damaging effects. Conversely, in 1G1A, stabilization between the corresponding structural elements is provided mainly by bonds between Tyr72 and Glu55 [14].

Ala89 in zebrafish and Ala100 in human TGDS are perfectly conserved across prokaryotes, plants, and higher animals (Fig. 2, green arrow). A closer structural examination revealed that these Ala residues occupy equivalent positions in zebrafish Tgds and human TGDS (Fig. 6B). Moreover, the corresponding Ala82 in the bacterial 1G1A enzyme is located near the nicotinamide ribose, with Tyr167 and Lys171 as part of the catalytic triad (Fig. S4). *In silico* mutagenesis of 1G1A Ala82 to Ser suggests the potential for new interactions, specifically between the polar Ser side chain and either the side chain of the highly conserved Asn100 (indicated by a green star in Fig. 2) or the hydroxyl groups of the nicotinamide ribose (Fig. S4). Therefore, within the limitations of this computational approach, the reduced activity observed for the Ala89Ser zebrafish mutant could be reasonably attributed to interference with the coenzyme‐binding pocket and improper NAD^+^ positioning. Finally, Tgds Tyr223 is again highly conserved throughout evolution, from bacteria (Tyr235 in 1G1A) to Tyr234 of the human protein (Fig. 2, green arrow, and Fig. 6C). In the WT protein, Tyr223 can form a π‐π interaction with the Tyr186 edge and hydrophobic interactions with Val307, which are also conserved in human TGDS (Tyr197 and Val318). The substitution of Tyr with His could impact the Tyr aromatic interactions, explaining the lower stability of this mutant. Taken together, these results are consistent with those obtained from the enzymatic analyses and support the damaging effects of the substitutions found in CMS patients. Interestingly, the predictions of AlphaMissense on the human TGDS substitutions categorized Ala100Ser and Tyr234His as likely benign, whereas Glu90Gly was uncertain, confirming some actual limits of this model to predict effects on protein stability [26].

### Effects of *tgds* knockout on F_0_ zebrafish development

CRISPR/Cas9‐mediated F_0_ knockout was subsequently employed to establish the developmental effect of *tgds* loss of function in zebrafish. This step aimed to obtain initial evidence supporting zebrafish as a suitable model for identifying the functional role of *tgds in vivo* and elucidating the biochemical and molecular defects underlying CMS pathogenesis. The generation of F_0_ biallelic knockouts through the simultaneous injection of multiple single guide RNAs (sgRNAs) can recapitulate complex phenotypes and has been utilized to rapidly screen for gene ablation effects [27].

**Fig. 7:**
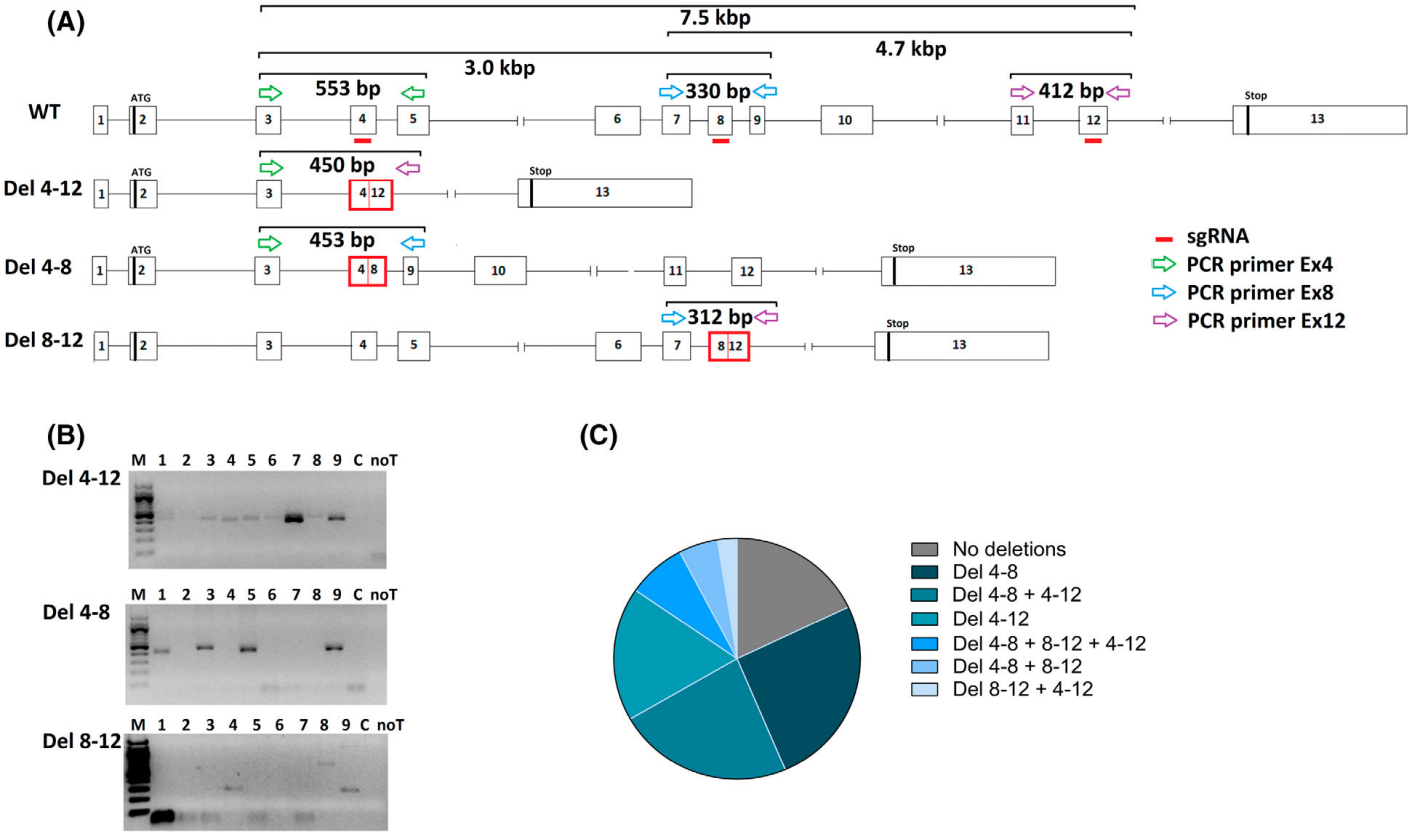
Genotyping strategy and results for F_0_ embryos after CRISPR‐Cas9 gene editing. (A) Scheme of the *tgds* gene and strategy used for detecting the generated large deletions. The figure shows the gene organization with exons and the specific exons targeted by the guide RNAs (sgRNAs), which are indicated in red. The diagram highlights the different primer combinations used in PCR to amplify DNA fragments only if a large deletion occurred between the targeted exons. For example, primers spanning exons 4 and 12 would only produce a smaller amplicon if the intervening exons (5–11) were deleted. This strategy allows the detection of specific deletion events (Del4‐12, Del4‐8, and Del8‐12). (B) Representative genotyping results from nine embryos injected with the target sgRNAs, following the strategy outlined in panel A. The observed amplicon sizes agree with the expected size. Specifically, embryos #1, #3, and #5 presented both Del4‐12 and Del4‐8. Embryo #4 included Del4‐12 and Del8‐12. Notably, embryo #9 displayed all three deletion types. The control C, which was injected with a scrambled sgRNA, and the no‐template control, noT, showed no amplification. (C) Frequencies of deletions in 40 genotyped embryos obtained across three independent microinjections. Approximately 80% of the embryos presented at least one deletion. Del8‐12 was never found as an isolated deletion. Del4‐12 and Del4‐8, either isolated or in combination, accounted for most cases.

Three sgRNAs targeting exons 4, 8, and 12 were designed and injected into one‐cell‐stage embryos. This strategy aimed to induce large deletions within the *tgds* gene that encompass several exons and abolish protein function. Figure 7A illustrates the sgRNA target regions and the PCR strategy implemented to detect the presence of these large deletions. Indeed, the utilization of various combinations of PCR primers and a short elongation step yielded smaller amplicons only when a specific deletion was present, namely, the deletions between exons 4 and 12 (Del4‐12), between exons 4 and 8 (Del4‐8), and between exons 8 and 12 (Del8‐12). Detailed information regarding the sgRNAs and PCR primers is provided in Table S1.

Genotyping of F_0_ embryos by PCR (Fig. 7B) revealed the presence of the expected deletions, which were subsequently analyzed by Sanger sequencing of selected amplicons (Fig. S5), confirming the validity of the approach. Genotyping of 50 embryos (Fig. 7C) revealed that approximately 80% exhibited at least one large deletion, demonstrating the efficiency of the editing strategy. Notably, large deletions were present in 100% of the embryos with gross malformations. While Del4‐8 and Del4‐12 frequently co‐occurred, Del8‐12 was less common and was never found as an isolated deletion. The rare coexistence of all three deletions in some embryos confirmed mosaicism (Fig. 7C). As small insertions or deletions within each exon were not investigated, the potential contribution of other editing events to the mosaic genotype cannot be excluded.

**Fig. 8:**
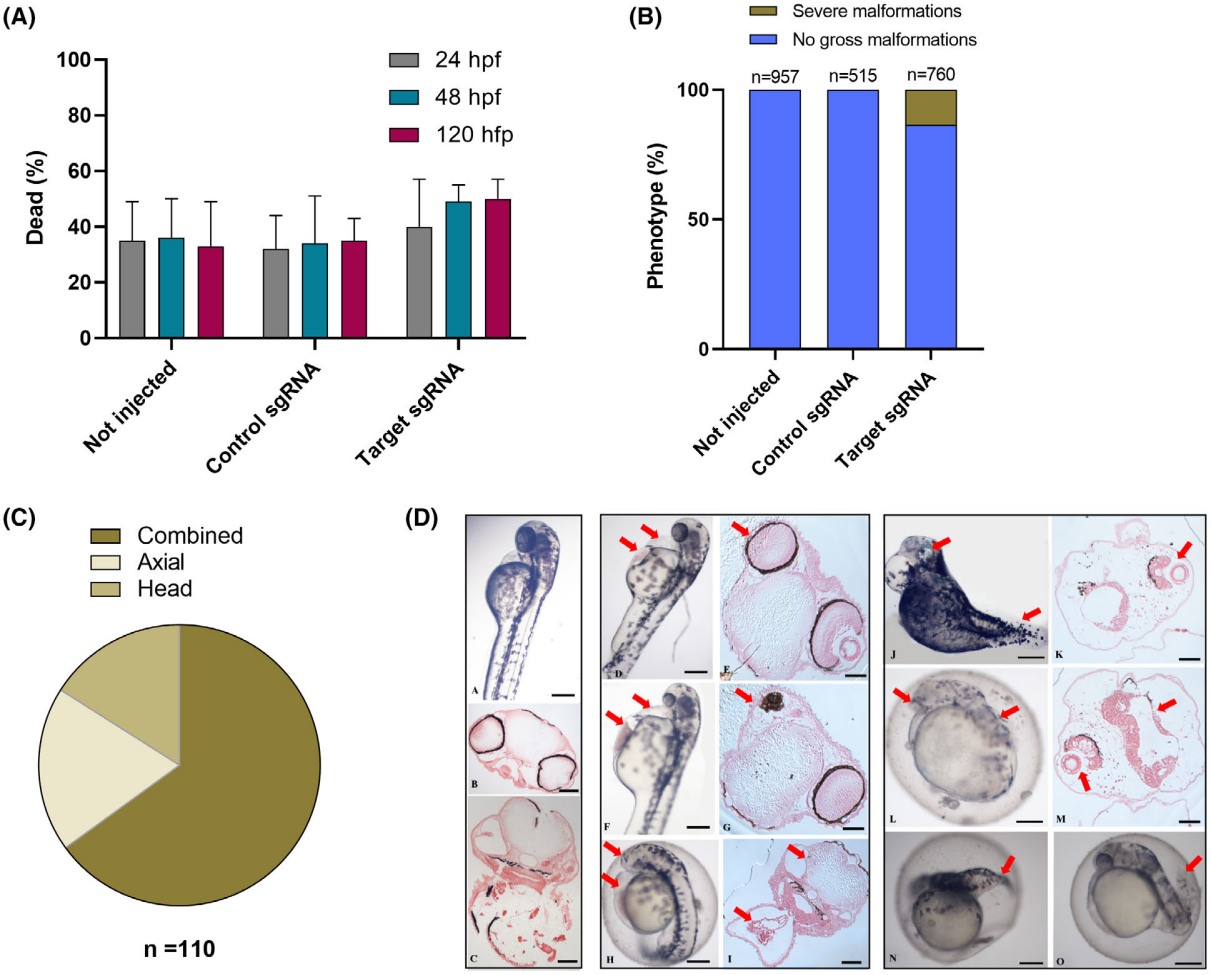
Mortality and phenotypic analysis of F_0_ embryos. (A) Embryo mortality rates were tracked at various hours postfertilization (hpf) for noninjected embryos and those microinjected with either scramble or target sgRNAs. The data, compiled from four independent microinjections, revealed variability in mortality across different clutches. However, the differences in mortality rates between the experimental groups did not reach statistical significance (mean ± SD, one‐way ANOVA). The total number of embryos analyzed was as follows: not injected, *n* = 1796; control sgRNA, *n* = 1030; and target sgRNAs, *n* = 1642. (B) Daily observation of embryos identified individuals with evident gross malformations, which were subsequently euthanized. This panel presents the cumulative percentage of embryos exhibiting severe phenotypes at 120 hpf, derived from three independent microinjections. The total number of embryos inspected for each group is indicated above the respective bars. (C) The severe malformations observed in embryos injected with target sgRNAs, as quantified in panel B (*n* = 110), are categorized into three main types: (a) head malformations, including instances of absent or duplicated heads and small or absent eyes. (b) Axial malformations: characterized by a lateral curvature of the body axis. (c) Miscellaneous: represents the largest category, encompassing embryos exhibiting multiple severe malformations occurring in combination. (D) Representative images illustrate severely malformed embryos (D to O) in comparison to control embryos (A–C). Edema accompanied by blood accumulation in the pericardial region (highlighted by the red arrows in panels D, F and H) is associated with abnormal eye (red arrows in panels E and G) and otic vesicle development and blood accumulation (red arrows in panel I). Severely impaired head and body development is highlighted by the red arrows in panels J and L (and corresponding sections in panels K and M). Abnormal axial development (red arrows) is observed in panels N and O. Scale bars are 100 μm for whole mounts and 50 μm for sections.

The embryo mortality rates following injection with the three target RNPs were slightly higher compared to the noninjected embryos or embryos injected with a scrambled control, but the difference did not reach statistical significance (Fig. 8A). Initial microscopic examination revealed severe morphological abnormalities in approximately 13% of the surviving embryos, already evident between 48 and 72 hpf (Fig. 8B). These aberrant phenotypes, summarized in Fig. 8C and illustrated as examples in Fig. 8D, often co‐occur in the same embryo. They included gross malformation of the body shape, aberrant head and eye development, a curved axis, blood accumulation, and edema in the pericardial region. Abnormal development of the eyes and abnormal axial growth were the most frequently observed alteration in the combined phenotypes. Interestingly, most of the severe malformations observed in crispants are also found in *tgds* null mouse embryos from the IMPC (https://www.mousephenotype.org/) [28]. Representative images for *tgds* knockouts at E9.5 are presented in Fig. S6, which include individuals with gross malformation of the body shape, abnormal heart and neural tube morphology, abnormal embryo turning, head and optic vesicle underdevelopment, alterations in the somite shape, and blood accumulation in some body regions. Abnormal testis, eye, and kidney morphology are also reported for the heterozygous adults.

**Fig. 9:**
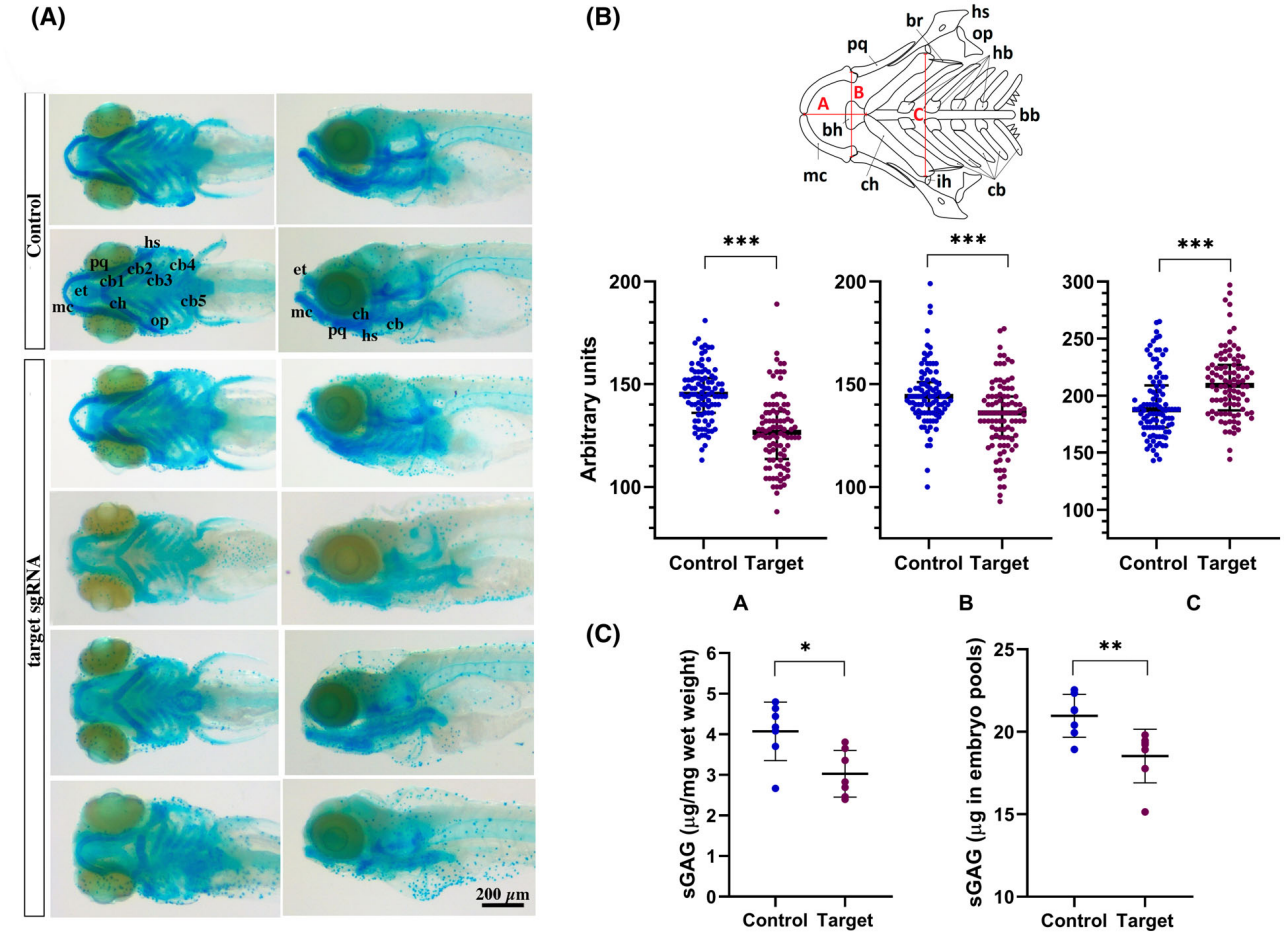
Alcian blue staining of cartilage and sGAG quantification. At 120 hpf, embryos without gross morphological alterations (as shown in Fig. 8B) underwent Alcian blue staining and whole‐mount stereomicroscopic analysis. (A) Ventral and lateral views revealed a spectrum of cartilage malformations in the sgRNA‐injected embryos (four lower panels) compared with the control embryos (two upper panels). These defects range from near‐normal cartilage to significant disruption of craniofacial structures. Cartilages are identified with the following abbreviation: bb, basibranchials; bh, basihyal; cb, ceratobranchial; ch, ceratohyal; hb, hypobranchial; hs, hysosymplectic; ih, interhyal; mc, Meckel's cartilage; op, opercle; pq, palatoquadrate. Scale bar = 200 μm. (B) A schematic of the 120 hpf zebrafish viscerocranium (ventral view) illustrates the cartilages analyzed for the morphometric measurements (red lines). The abbreviations are the same used in panel A. Morphometric analysis revealed a significant reduction in the development of Meckel's cartilage and the palatoquadrate (measurements A and B) and a wider angle between the ceratohyals (measurement C) in most injected larvae. Analysis was performed by Mann–Whitney test, *n* = 96 per group, ***P < 0.001; median with interquartile range is depicted. (C) Analysis using the DMMB colorimetric assay reveals a significant reduction in sulfated glycosaminoglycans (sGAG) levels in the embryos injected with the target sgRNA. Pools of 10 embryos each from control and targeting sgRNA‐injected animals were extensively digested with papain, and the released sGAG were quantified against a heparan sulfate standard curve. Results are expressed as μg of sGAG for mg of wet weight or as total sGAG content in the embryo pools. Analysis was performed by Welch *t*‐test, *n* = 8 per group, *P < 0.05; **P < 0.01; mean ± SD is depicted.

By 120‐h postfertilization (hpf), most embryos injected with the target RNPs presented additional subtle malformations, particularly in terms of head morphology. Alcian blue staining was subsequently performed on these larvae to examine head cartilage morphology in detail. This revealed distinct alterations in craniofacial cartilage structure in embryos injected with the target RNPs compared with controls (Fig. 9A). The malformations displayed different severities, ranging from almost normal structures to more severe abnormalities. More examples of representative affected phenotypes compared with controls are reported in Fig. S7. Structures that form the lower jaw, specifically Meckel's cartilage, are significantly underdeveloped. Moreover, the cartilages derived from the second arch (i.e., ceratohyal, basihyal, and hyosymplectic) were distorted, and the ceratobranchials were disorganized. Morphometric analysis performed on the viscerocranium (Fig. 9B) confirmed a significant reduction in measurement A, which was related to Meckel's cartilage and the palatoquadrate cartilage length, and in measurement B, which was determined by Meckel's cartilage development. A wider angle between the ceratohyal cartilages (measurement C) was consistently found. Furthermore, the ethmoid plate was also affected in a subset of injected embryos, as particularly evidenced in the lateral views (Fig. 9A, Fig. S7). This abnormal cartilage development appeared even more pronounced in some animals examined at 10 dpf (Fig. S7). Importantly, no apparent morphological alterations were observed in noninjected animals or embryos injected with the control RNP, thus excluding potential nonspecific effects of the injection procedure.

Notably, several CRISPR‐edited embryos had less intense Alcian blue staining in their cartilage, suggesting a lower proteoglycan content. We confirmed this by comparing the total sulfated glycosaminoglycan (sGAG) content between control and CRISPR‐edited embryos at 120 hpf using the 1,9‐dimethylmethylene blue (DMMB) colorimetric assay. Because of the small amount of sGAG per embryo, we pooled 10 embryos together, creating eight separate pools for both the control and CRISPR‐edited groups. This pooling strategy accounted for individual developmental variations. The CRISPR‐edited pools showed a slight but significant reduction in sGAG content compared to controls (Fig. 9C). This was true whether we measured the total sGAGs per pool of 10 embryos or normalized the sGAG content by the tissue's wet weight.

Our efforts to establish a stable *tgds* knockout line in zebrafish were so far unsuccessful, using both the three sgRNA approach or using a single sgRNA. We have faced significant challenges, including a high mortality rate (80–90%) in crispants between 10 and 15 days postfertilization (dpf), even for individuals not displaying overt defects. This high mortality may be linked to subtle craniofacial alterations that affect feeding and swim bladder inflation, or other less obvious organ malformations. Few individuals reaching adulthood either did not show any editing or, if showing the presence of deletions by fin‐clipping genotyping, they were unable to transmit them to the progeny to generate heterozygous animals. While null mice were successfully obtained by the IMPC, the heterozygous males are reported to have testis enlargement and malformations, suggesting that *tgds* haploinsufficiency impacts gonad development [28]. The differences in gonad development between fish and mammals may account for the difficulty in obtaining edited gametes from our crispants.

## Discussion

Although orthologs of TGDS are present in most vertebrate genomes, as we already reported [17] and further confirmed in this study, their function in these animals remains an open question. Our study provides the first evidence that zebrafish Tgds is a functional enzyme that catalyzes UDP‐D‐Glc dehydration, forming UDP‐KDG, the first step for the synthesis of L‐Rha and other 6‐deoxyhexoses, as already recognized in lower eukaryotes, giant viruses, and plants [15, 17, 18, 19, 24]. The crucial involvement of UDP‐D‐Glc in glycogen synthesis, UDP‐D‐galactose metabolism, and the formation of the essential proteoglycan components UDP‐D‐GlcA and UDP‐D‐Xyl strongly suggests that Tgds has access to its substrate *in vivo*. Importantly, since Tgds is an Uxs1 paralog, our results exclude a direct Tgds involvement in UDP‐D‐GlcA decarboxylation, forming UDP‐D‐Xyl. As expected for its eukaryotic origin, the enzyme activity showed a strong preference for the UDP‐bound substrate, with low activity on TDP‐D‐Glc, as we already demonstrated for the Mimivirus and *T. vaginalis* UGD enzymes [15, 17]. Specifically, zebrafish Tgds Gln211 residue could develop the same interaction with the ribose C‐2′ hydroxyl group of UDP observed for Gln206 of Mimivirus UGD or His214 of *T. vaginalis*, which stabilizes substrate binding [17].

The identification of Tgds enzymatic activity suggests that higher animals could produce another 6‐deoxyhexose in addition to the well‐known L‐fucose. While L‐fucose role in vertebrates is well established [29], the presence of L‐Rha or similar modified sugars in complex glycans is less documented [30]. However, the synthesis of ‘endogenous ouabain’ (OLC), a cardioactive compound containing rhamnose acting as a ligand of the Na^+^/K^+^‐ATPase, has been consistently reported [31]. Notably, OLC production was observed in cultured adrenocortical cells even without exogenous L‐Rha, indicating its endogenous synthesis [32]. This suggests that higher animals may possess an L‐Rha biosynthetic pathway used for producing active glycosides, as it happens in plants, that is, for the flavonoids and saponins [33]. Besides the formation of the 6‐deoxyhexose, other possible functions for the dehydratase activity can be envisioned. UDP‐KDG itself could have a direct effect, due to its structural similarity with nucleotide‐sugar‐utilizing enzyme substrates, influencing their activity. This has already been demonstrated in the pathogenic fungus *Botrytis cinerea*, where UDP‐KDG acts as an antifungal metabolite by competitively inhibiting UDP‐galactopyranose mutase [34]. Another intriguing possibility is that Tgds activity could affect the intracellular UDP‐D‐Glc pool. Wang *et al*. [35] indeed found that HuR interaction with UDP‐D‐Glc prevents its binding to and stabilization of SNAI1 mRNA. Depletion of UDP‐D‐Glc by UDP‐D‐Glc dehydrogenase (UGDH), which is activated by EGFR phosphorylation and subsequently binds to HuR, enables HuR to stabilize the SNAI1 transcript. This leads to SNAIL protein production, facilitating epithelial‐mesenchymal transition. Currently, the prevailing view is that human cells lack an NDP‐Glc 4,6‐dehydratase, making this activity a promising target for antibacterial drug development against L‐Rha‐producing pathogens [36, 37, 38]. Our findings, however, suggest a need for re‐evaluation and further research from this perspective.

The catalytic activity observed for Tgds does not necessarily represent the only function. It is possible that it acquired new properties over time, such as the ability to interact with other proteins, making it a ‘moonlighting’ protein. The ‘moonlighting’ proteins display more than one physiological function, that is, they can be both enzymes of a metabolic pathway and regulatory effectors [39]. Several members of the short‐chain dehydrogenase/reductase (SDR) superfamily display these properties, including UGDH as reported above. For instance, the GDP‐D‐mannose 4,6‐dehydrase (GMD), the first enzyme of the GDP‐L‐fucose pathway, is also a binding partner of human tankyrase‐1, a poly(ADP‐ribose) polymerase that affects telomere length, influencing its stability and activity [40]. HSD17B10, an SDR oxidoreductase acting on a wide range of substrates, from branched amino acids to steroids, is also ‘moonlighting’ as a component of the mitochondrial RNase P complex [41]. In any case, given the vast evolutionary distance between zebrafish and other eukaryotes where the dehydratase activity has been confirmed—such as *T. vaginalis* (1.5 billion years, estimated by TimeTree5, www.timetree.org) [17], fungi (1.3 billion years) [19], and plants (1.5 billion years) [18]—the conservation of this enzymatic activity is, in itself, a significant finding regarding protein evolutionary constraints.

We have also demonstrated the damaging effects of some of the substitutions observed in CMS patients on the zebrafish Tgds. Considering the high degree of structural conservation between the zebrafish Tgds and human TGDS proteins, it is reasonable to hypothesize that these corresponding mutations in humans would exhibit similar damaging effects, thereby providing insights into the biochemical basis of CMS. The Ala89Ser mutation in zebrafish had milder effects on both catalytic activity and stability; this substitution possibly interferes with enzyme activity by perturbing the position of NAD^+^ in the active site. The corresponding human Ala100Ser is considered a founder mutation [1], and it is the only mutation found in homozygosity in patients. On the other hand, the Glu79Gly and Tyr232His mutations had more severe consequences, strongly affecting protein stability; this finding was also supported by structural model analysis. Interestingly, these mutations (Glu90Gly and Tyr234His in TGDS) are found as compound heterozygous variants in CMS patients and are generally associated with the milder Ala100Ser [1, 5, 6]. Collectively, these data represent the first biochemical demonstration of mutation effects, providing further support for the involvement of TGDS in the pathogenesis of CMS, as proposed by Ehmke *et al*. via genetic studies [1].

Zebrafish *tgds* gene is located in the subtelomeric region of chromosome 6 and consists of 13 exons. The position of the *tgds* locus at the telomeric region not only poses challenges in targeting and cloning but also raises several interesting questions about the possible implications. These regions are known to be rich in repetitive elements, less stable, and prone to silencing, positional effects, or even telomeric shortening. Conversely, the human gene is formed by 12 exons in chromosome 13q32.1, which is a more internal region than the zebrafish gene, as it can also be observed for other vertebrates. Notably, the absence of a paralog, an otherwise common feature due to teleost whole‐genome duplication, simplifies the CRISPR/Cas9‐mediated knockout approach.

To further confirm that zebrafish are a relevant *in vivo* model for deciphering CMS pathogenesis, we investigated the consequences of *tgds* loss of function via CRISPR/Cas9‐mediated F_0_ knockout. This study revealed a range of developmental defects of varying degrees of severity. A small subset of embryos displayed pronounced abnormalities, particularly affecting the head and axial skeleton development. Alterations in the eyes and otic vesicles were also found, in agreement with *tgds* expression in these regions determined by ISH. Interestingly, preliminary data from the IMPC [28] on the *tgds* null mice reveal early embryonic lethality with complete penetrance, with severe phenotypes resembling some of the features found also in the F_0_ zebrafish embryos reported in Fig. 8. Even if the developmental stages of the displayed zebrafish embryos (48–72 hpf) and the mouse embryos are not the same (with E9.5 in the mouse roughly corresponding to pharyngula stage between 24 and 48 hpf in the zebrafish), these findings from both organisms highlight that *tgds* is an essential gene and plays a key role for the correct development in vertebrates. They can also explain the difficulties we are experiencing in obtaining a stable knockout line in zebrafish.

With the CRISPR/Cas9 protocol and the conditions used for the present study, most of the zebrafish embryos injected with the target RNPs did not show gross evident malformations, but they presented significant alterations in craniofacial cartilage development, more resembling the hypomorphic phenotype observed in CMS patients. The apparent discrepancy between the two groups of embryos, in part also observed in the null mice, can be explained by developmental adaptation in some animals, the occurrence of compensatory mechanisms, and for the crispants, the presence of the genetic mosaicism that is inherent in the procedure. In particular, the yolk injection used in this study could result to less consistent biallelic knockouts compared to the cytosolic injections [42]. The mosaicism can lead to diverse outcomes depending on the proportion of knockout cells within each individual, the potential loss of edited cells with lower fitness, and compensatory mechanisms arising from nonedited cells [43]. In this perspective, the severe alterations could be related to a prevalent biallelic knockout occurring in the zygote or during the first divisions and affecting the majority of the cells. On the other hand, a more extensive genetic mosaicism could explain the hypomorphic effects. Notably, among the craniofacial alterations observed in most of the injected embryos, the defects in Meckel's cartilage and the ethmoid plate mirrored some of the skeletal issues observed in CMS patients, specifically lower jaw hypoplasia and cleft palate, suggesting that our model can be useful to study *tgds* role in the development of facial cartilages. The lower intensity of Alcian blue staining observed in the crispants is also suggestive of a reduced proteoglycan content, which was confirmed by the sGAG analysis. However, it is not yet clear whether an altered proteoglycan production represents a cause of the phenotypic alterations, as previously suggested [11], or a consequence of defective neural crest‐derived cell migration and differentiation. Indeed, the key role of proteoglycans, such as glypicans and their heparan sulfate chains, in the control of Shh, Wnt, and Bmp signaling, altogether factors affecting cartilage development, is well‐known [44]. However, facial skeleton development is very complex, and a multitude of other pathways govern this process that could be affected by *tgds* ablation. Interestingly, we did not find *tgds*' orthologs in the jawless fishes, which present a specific embryonic morphological pattern (the so‐called ‘cyclostome pattern’) of craniofacial development [45], suggesting a specific role of *tgds* in gnathostomes.

The differing phenotypes between the two groups of crispants (severe vs. milder) suggest that a complete knockout stable line, while challenging to develop, may not be the most suitable model for studying the events leading to the clinical presentation in CMS patients. This is because the complete loss of *tgds* could have such a severe impact on major developmental processes that it doesn't accurately reflect the milder, hypomorphic defects seen in the disease. Therefore, we believe that a knockdown strategy or a knock‐in approach incorporating the specific mutations identified in our study would be more effective at recapitulating the disease's phenotype and could provide a reliable model for analyzing CMS.

Another important aspect that is highlighted by this study is the potential role of maternally derived *tgds* transcripts. Indeed, we observed that *tgds* is expressed at the highest levels in the zygote, followed by a significant decrease during the first cellular divisions, a pattern typical of the maternal effect genes. Then, the second wave of *tgds* transcription is initiated from the zygotic genome during somitogenesis, occurring after major zygotic genome activation (ZGA) at the mid‐blastula transition (approximately the 10th division, 3–3.5 hpf) [46]. Notably, a similar biphasic expression pattern, characterized by maternal contribution followed by later zygotic expression, has also been reported in transcriptomic profiles from frogs, suggesting that this expression dynamic is a conserved feature among vertebrates. Moreover, these findings indicate that *tgds* might play an important role in the very initial phases of embryo development. Our CRISPR/Cas9‐mediated gene ablation in F_0_ embryos targets the zygotic genome, and the resulting phenotypes are expected to reflect primarily the loss of *tgds*' second wave of transcription, which starts at the end of the gastrula stage. From this perspective, CRISPR/Cas13 represents an alternative tool for investigating the role of maternally derived genes in early development. Cas13 is an RNA‐guided RNA endonuclease that can target and degrade maternal mRNA transcripts present in early embryos, enabling the study of their function before the onset of zygotic gene expression; recently, its use has been optimized to prevent collateral effects [47].

In conclusion, we have biochemically validated the UDP‐D‐Glc dehydratase activity of zebrafish Tgds, a function conserved from that of lower eukaryotes, highlighting its potential involvement in a pathway still unrecognized in vertebrates. Our characterization of disease‐associated mutations in zebrafish Tgds provides the first functional evidence supporting their damaging effects and offers insights into the molecular basis of human CMS. Moreover, the observed craniofacial cartilage defects strikingly parallel skeletal abnormalities in CMS patients, further underscoring zebrafish as a valuable *in vivo* model for understanding Tgds function *in vivo* and unraveling the complexity of CMS pathogenesis.

## Materials and methods

### Bioinformatic analyses

The *tgds* gene and transcript sequences are obtained from GenBank. Protein sequence alignments were performed with Espresso on the T‐coffee server (https://tcoffee.crg.eu/), and figures were generated via ESPript (https://espript.ibcp.fr/ESPript/ESPript/) [48]. The predicted change in stability of the mutants was obtained via DynaMut2 (https://biosig.lab.uq.edu.au/dynamut2/) [25]. Structural models were retrieved from the AlphaFold protein structure database (https://alphafold.ebi.ac.uk/) [49]. AlphaMissense prediction were obtained from https://alphamissense.hegelab.org/ [26].

### Zebrafish care and breeding

*Danio rerio* (zebrafish) wild‐type line of the Tuebingen strain used in this study was bred in the zebrafish facility of the Department of Earth, Environment, and Life Sciences, University of Genova. All experiments were performed in compliance with Italian and European legislation (Legislative Decree 26/2014 and Directive 2010/63/EU) and following authorization from the Ethics Committee of the University of Genova and the Italian Ministry of Health (Authorization number no. 384/2023‐PR).

Adult zebrafishes were maintained in a commercial recirculating system (Tecniplast, Buguggiate, Italy) at 28.5 °C and subjected to a 14 : 10 light–dark cycle as previously described [50]. Animals were fed granular food three times a day (Zebrafeed, Sparos Ltd., Olhão, Portugal) and *Artemia* nauplii twice a week. Embryos were obtained via natural mating according to standard procedures [50]. The embryos were raised in Petri dishes at 28.5 °C until the desired stages were reached according to Kimmel *et al*. [51]; the embryos destined for *in situ* hybridization analyses were raised in the presence of 0.003% (wt/vol) 1‐phenyl‐2‐thiourea to prevent pigment formation. For organ explants, adult samples were euthanized by MS222 overdose followed by beheading.

### RNA extraction and cDNA synthesis

Embryos at the desired stage after euthanasia and organs were homogenized in TRI reagent (Merck Life Science, Milan, Italy). RNA was extracted following the manufacturer's instructions and further purified via the RNeasy Mini Kit (Qiagen, Hilden, Germany) according to published protocols [52]. For all the samples, cDNA was synthesized via the SuperScript III First‐Strand Synthesis System with oligo(dT) primers (Thermo Fisher Scientific, Waltham, MA, USA).

### Analysis of transcript expression

Taq DNA polymerase and dye‐based Luna Universal qPCR Master Mix (both from New England Biolabs, Ipswich, MA, USA) were used for PCR and RT–qPCR, respectively, following the manufacturer's protocols. The custom primers (Invitrogen) used for amplification are reported in Table S2. For RT–qPCR, the $2^{-\Delta \Delta C_{t}}$ method was applied, with *actb2* and *eef1a used* as housekeeping genes [53, 54], which gave comparable results; the reference conditions used are indicated in the figure legends. Statistical analyses (Welch's *t* test) were performed in at least triplicate experiments via graphpad prism 8.0 (GraphPad Sofware, Boston, MA, USA).

Publicly available RNA‐seq data of zebrafish developmental stages were obtained from the EBI European Nucleotide Archive (accession no: PRJEB12982) [22]. The downloaded Fastq files were aligned via starv2.7 [55] (RRID:SCR_004463) to a modified version of the GRCz11 *Danio rerio* genome assembly, which contains custom annotations for the *tdgs* gene and corresponding transcript. The transcripts were quantified via FeatureCounts [56] (RRID:SCR_012919). Normalization and visualization of the gene expression data were performed via r programming (https://www.R‐project.org/) [57] (RRID:SCR_001905) and the tidyverse package5 [58] (RRID:SCR_019186).

### Whole‐mount *in situ* hybridization, sectioning and imaging

The plasmid containing the complete coding sequence of *tgds* was linearized by the restriction enzyme Not*I* and used to synthesize a DIG‐labeled antisense riboprobe via an RNA labeling kit (Roche Applied Science, Penzberg, Germany) according to the manufacturer's recommendations. A sense riboprobe was produced as a negative control.

Embryos at the desired stages were fixed in 4% paraformaldehyde (Merck Life Science) in PBS buffer (0.8% NaCl, 0.02% KCl, 0.02 m PO_4_, pH 7.3) at +4 °C overnight, subsequently dehydrated in a graded methanol series, and stored in 100% methanol at −20 °C for *in situ* hybridization analyses. Whole‐mount *in situ* hybridization was performed as previously reported [59, 60], and the embryos were imaged via an IX71 inverted microscope equipped with a ColorView II camera (Olympus, Hamburg, Germany). The selected embryos were then counterstained with 1% Ponceau S, embedded in Spurr's resin (Merck Life Sciences), and serially sectioned at 4 μm [60].

### Protein expression and site‐directed mutagenesis

Zebrafish cDNA was obtained from purified ovary RNA as described in the ‘RNA extraction and cDNA synthesis’ section. The *tgds* coding region was amplified via PCR via *Q5* high‐fidelity polymerase (New England Biolabs); the primers used are listed in Table S2. The PCR product was purified (PCR clean‐up kit, Merck Life Science) and digested with NdeI and XhoI. The pET‐28a vector (Merck Life Science) was doubly digested with the same enzymes and dephosphorylated by Antarctic phosphatase; ligation was performed via a Quick Ligation Kit following the indicated protocol. All reagents used for cloning were obtained from New England Biolabs. The construct was sequenced (BMR Genomics, Padova, Italy) and transformed into *E. coli* BL21 DE.

For protein expression, the cells were grown at 18 °C in 2XYT medium; isopropyl β‐D‐1‐thiogalactopyranoside was added at a final concentration of 0.1 mm when the culture *A*_600_ reached 0.6. After induction, the cells were further incubated overnight at 18 °C. The protein containing the N‐terminal 6xHis tag was purified via ProBond™ Nickel‐Chelating Resin (Thermo Fisher Scientific) under native conditions according to the manufacturer's instructions. Elution was achieved via the use of 250 mm imidazole. All the purification steps were performed at 4 °C. The eluted fractions were concentrated via the centrifugal filter Amicon® Ultra (Merck Life Sciences). The protein concentrations were determined via UV spectrophotometry at 280 nm via a calculated extinction coefficient of 56 389 m ^−1^ cm^−1^ via the ProtParam tool on the ExPASy server [61]; the purity was determined via SDS/PAGE.

Site‐directed mutagenesis was performed via the Quickchange II system (Agilent, Santa Clara, CA, USA). The mutagenic primers used are listed in Table S2. The correct substitutions were confirmed by sequencing (BMR Genomics); the mutant proteins were then expressed and purified as described above.

### Enzymatic analysis

The enzymatic activity of the expressed proteins was determined by an HPLC assay. In a standard assay, purified Tgds was incubated at 30 °C in PBS (pH 7.3) with 200 μm of the following substrates (Merck Life Science): UDP‐D‐Glc, UDP‐D‐galactose, UDP‐D‐GlcA, and UDP‐N‐acetyl‐D‐glucosamine. dTDP‐D‐Glc was produced in‐house as previously described [62]. In some experiments, NAD(P)^+^ (100 μm) and bivalent cations (5 mm MgCl_2_ or MnCl_2_) were added. At various time points, aliquots were removed, and the protein was precipitated by heating at 80 °C for 3 min, followed by centrifugation. HPLC analysis was performed using a Wesca/R anion exchange column with a 100 μL injection volume. The peak retention time of UDP‐KDG was compared in parallel injections with that obtained using T. vaginalis UDP‐D‐Glc 4,6‐dehydratase (EC4.2.1.46) [17]. The identity of the dehydration reaction product was confirmed by ESI–MS directly on the reaction mixture without purification of the compounds, as previously described [15, 17]. The amount of NAD^+^ tightly bound to the protein was quantified using a cycling assay, as previously reported [63], and the coenzyme/protein monomer ratio was determined via the calculated protein extinction coefficient. Protein stability was assessed by incubating purified proteins at 1 mg·mL^−1^ in PBS at 4 °C for several days. At specific time points, the protein was centrifuged at 12 000 ***g*** for 10 min, and the decrease in absorbance at 280 nm in the supernatant was measured.

### CRISPR/Cas9‐mediated F_0_ knockout

Guide RNAs were designed through the CHOPCHOP program [64]. As *tgds* is not annotated in GRCz11, the exon sequences were manually inserted. On the basis of scoring efficiency and low predicted off‐target effects, three gRNAs targeting exons 4, 8, and 12 were selected, as detailed in Table S1. The targeted genomic regions were initially PCR amplified and Sanger sequenced to verify correct guide pairing. The primers used for PCR amplification are listed in Table S1. TrueCut™ Cas9 Protein v2 (5 μg·μL^−1^) and the sgRNAs were obtained from Thermo Fisher. Ribonucleoprotein particles (RNPs) were formed by mixing 1 μL of Cas9 protein (30 μm) with 1 μL of each target‐specific sgRNA (30 μm in RNase‐free water) and incubating at 37 °C for 5 min. Equal volumes of the three target RNPs were then mixed and immediately injected into the yolk of one‐cell‐stage embryos (volume of approximately 1 nL). For the control RNP, 3 μL of the nontargeting ‘scramble’ sgRNA (30 μm) was mixed with 3 μL of Cas9 protein and incubated under the same conditions.

Injections into the yolk of one‐cell‐stage embryos were performed via FemtoTip II capillaries (Eppendorf, Milan, Italy), an MP‐1 micromanipulator (Narishige Group, London, UK), and a FemtoJet® microinjector (Eppendorf, Milan, Italy) as previously described [65].

Following microinjection, the embryos were maintained overnight at 28 °C in fish water until 5 dpf. Phenotyping of the zebrafish embryos was conducted daily via a Leica stereomicroscope to determine the mortality rate and the development of gross malformations. After euthanasia, some of the embryos were used for genotyping, while the others were fixed with 4% PFA in PBS at 4 °C overnight and washed three times with PBS before further processing.

For genotyping, genomic DNA (gDNA) was extracted from individual embryos at 72 hpf via proteinase K digestion. Briefly, embryos were incubated in 20 μL of lysis buffer (10 mm Tris/HCl, pH 7.8; 1 mm EDTA; 0.3% NP‐40; 0.3% Tween 20; 5 mm CaCl_2_; and 10 μg·mL^−1^ proteinase K) for 3 h at 55 °C. Proteinase K was then inactivated by heating the samples at 99 °C for 10 min. PCR was performed directly on 10‐fold diluted extracts via various primer pairs (Thermo Fisher) to detect exons and deletions, as outlined in Fig. 7A and Table S1. Amplification was carried out via DreamTaq DNA polymerase (Thermo Fisher) according to the manufacturer's instructions. The PCR products were visualized on 2% agarose gels. Three amplicons for each identified deletion were subjected to Sanger sequencing to confirm gene editing (BMR Genomics).

Gross morphological alterations were inspected at 48 and 72 hpf via an IX71 inverted microscope equipped with a ColorView II camera (Olympus). Some embryos were then counterstained with 1% Ponceau S, embedded in Spurr's resin, and serially sectioned at 4 μm as previously described for hybridized embryos.

Alcian blue staining of 120 hpf embryos was performed under acidic conditions. Briefly, PFA‐fixed embryos were initially washed 3 times with PBS‐0.1% Tween (PBS‐T); then, the embryos were bleached with a solution of 3% H_2_O_2_ and 1% KOH in water for 10 min, followed by three 10‐min washes with PBS‐T. The staining solution consisted of 0.1% Alcian blue (Thermo Fisher) in 70% ethanol/1% HCl and was stirred for 1 h before paper filtration. Staining was conducted overnight at room temperature with continuous agitation. Destaining was performed using a 70% ethanol/5% HCl solution at room temperature for 4 h, with hourly solution changes. The embryos were then washed 3–4 times with PBS‐T for 5 min and transferred to 80% glycerol for imaging. Imaging was performed using a Leica EZ4 stereomicroscope (Leica, Wetzlar, Germany), keeping constant magnification and camera resolution. The distances between the cartilages, as indicated in Fig. 9B, were measured via imagej (https://imagej/net/software/fiji/).

Sulfated glycosaminoglycans (sGAGs) were analyzed using the Blyscan™ kit (Biocolor, Belfast, UK), following the supplier's instructions. Briefly, the assay is based on 1,9‐dimethylmethylene blue dye, which specifically binds to the sulfate polysaccharide component of proteoglycans. Analysis was performed using 8 pools of 10 embryos at 120 hpf, either controls or injected with the three targeting sgRNAs. All the water was carefully removed from the samples using a fine needle before measuring the wet weight of the embryo pools. Total sGAGs were extracted by papain digestion and analyzed accordingly to the instructions provided with the kit. The reference standard was bovine tracheal chondroitin 4‐sulfate.

Data were collected from three to four independent microinjections. graphpad prism 8.0 was used for all the statistical analyses; details are provided in the legends to the figures.

## Conflict of interest

The authors declare no conflict of interest.

## Author contributions

MRC: performed the experiments and analyzed the data. DB: performed the experiments and analyzed the data. EA: performed the experiments and analyzed the data. VB: performed the experiments. GC: performed the experiments. MB: analyzed the data. DC: analyzed the data. FP: provided reagents and analyzed the data. CP: performed the experiments. CW: planned experiments and reviewed the manuscript. SC: planned experiments, curated the data, and wrote the original draft. MT: planned the study, provided funding, planned experiments, curated the data, and wrote the original draft.

## Supporting information


**Fig. S1.** *tgds* expression during development in *X. tropicalis* and *X. laevis*.
**Fig. S2.** Zebrafish embryos hybridized with the sense probe as negative controls.
**Fig. S3.** Structural alignments of zebrafish Tgds with the human and bacterial orthologs.
**Fig. S4.** Close view of the coenzyme binding pocket of *S. enterica* 1G1A and the predicted Ala82Ser mutant.
**Fig. S5.** Representative results obtained from Sanger sequencing of the PCR products derived from genotyping.
**Fig. S6.** Representative images for WT and *tgds* knock‐out mice obtained from the International Mouse Phenotyping Consortium.
**Fig. S7.** Alcian blue staining of further examples for controls and target sgRNA injected embryos.
**Table S1.** sgRNA and PCR primers for CRISPR‐Cas9 and genotyping.
**Table S2.** List of primers for RT‐qPCR and cloning.

## Data Availability

The data that support the findings of this study are available in the figures, table, and supporting material of this article. Raw data are available upon request from the corresponding author (tonetti@unige.it).
