# Supplementary material for: Zebrafish as a model for Catel–Manzke syndrome—identification and characterization of the zebrafish TGDS ortholog
Source: FEBS J. 2025 Oct 29;293(8):2248–69. doi: 10.1111/febs.70307 (PMC13080230; doi:10.1111/febs.70307)
Supplement: Supplementary file 1 — Fig. S1. tgds expression during development in X. tropicalis and X. laevis. Fig. S2. Zebrafish embryos hybridized with the sense probe as negative controls. Fig. S3. Structural alignments of zebrafish Tgds with the human and bacterial orthologs. Fig. S4. Close view of the coenzyme binding pocket of S. enterica 1G1A and the predicted Ala82Ser mutant. Fig. S5. Representative results obtained from Sanger sequencing of the PCR products derived from genotyping. Fig. S6. Representative images for WT and tgds knock‐out mice obtained from the International Mouse Phenotyping Consortium. Fig. S7. Alcian blue staining of further examples for controls and target sgRNA injected embryos. Table S1. sgRNA and PCR primers for CRISPR‐Cas9 and genotyping. Table S2. List of primers for RT‐qPCR and cloning. [file FEBS-293-2248-s001.pdf]

Supporting Figure S1

A

Xenopus tropicalis

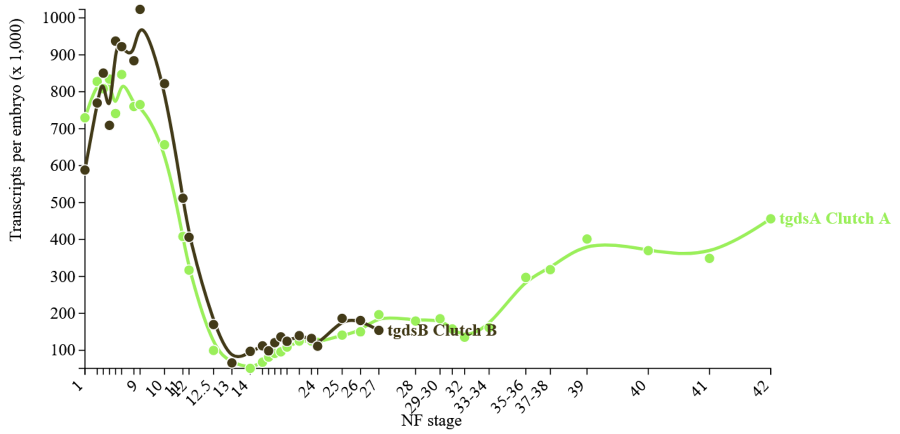

Xenopus laevis

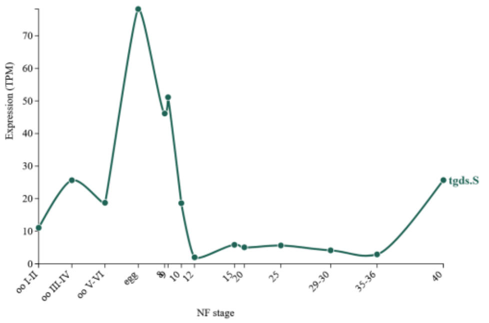

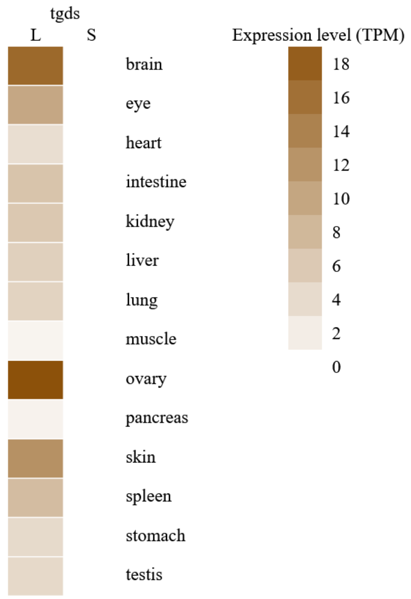

Figure S1. ***tgds*** **expression in** ***Xenopus tropicalis*** **and** ***Xenopus laevis*****.**
Upper panel: expression in *X. tropicalis* during development. Lower panel, left: expression in *X. laevis* during development; lower panel, right: expression in *X. laevis* adult tissues.
NF stages: stage 1, egg after fertilization; stage 9, late blastula; stages 10-12.5, gastrula; stages 13-21, neurola; stages 22-28, tailbud; stage 40, free swimming tadpole.
Data are from Xenbase (https://www.xenbase.org) (22).

Supporting Figure S2

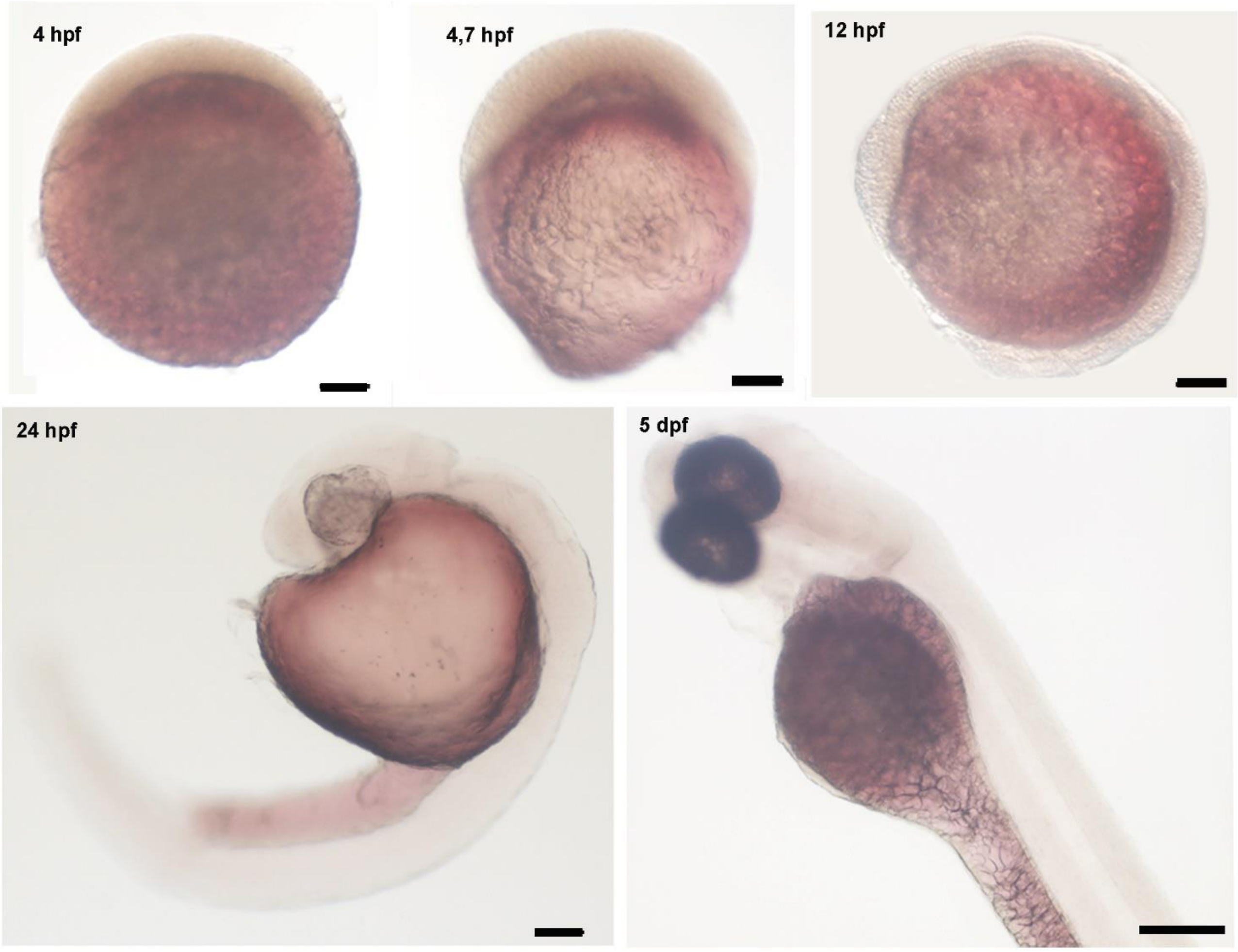

Figure S2. Zebrafish embryos hybridized with the sense probe as negative controls.
Scale bars are 100 µm.

Supporting Figure S3

A

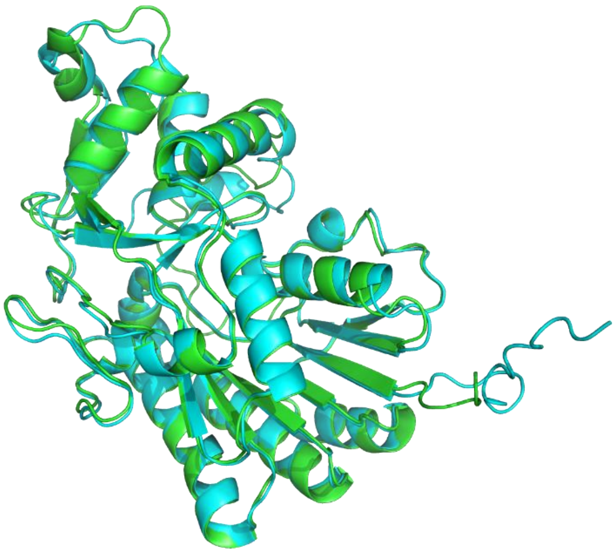

B

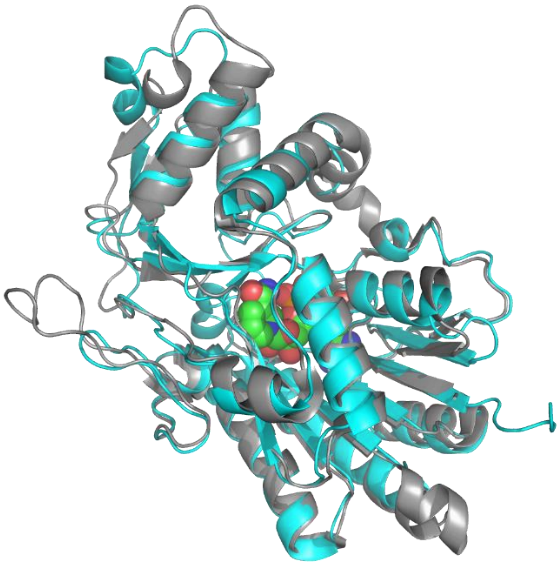

Figure S3. Structural alignments of zebrafish Tgds with the human and bacterial orthologs.
(A) Structural alignment of zebrafish Tgds (cyan) and human TGDS (green) Alphafold models. (B) Structural alignment of zebrafish Tgds (cyan) and *Salmonella enterica* serovar Typhimurium (gray). The NAD^+ molecule is visible in the crystallized bacterial enzyme. Images were obtained using PyMOL.
Zebrafish Tgds: Uniprot Q6NYF5, AlphaFold AF-Q6NYF5-F1-v4. Human TGDS: UniProt O95455, Alphafold AF-O95455-F1-v4. *S. enterica* RmlB: pdb 1G1A.

Supporting Figure S4

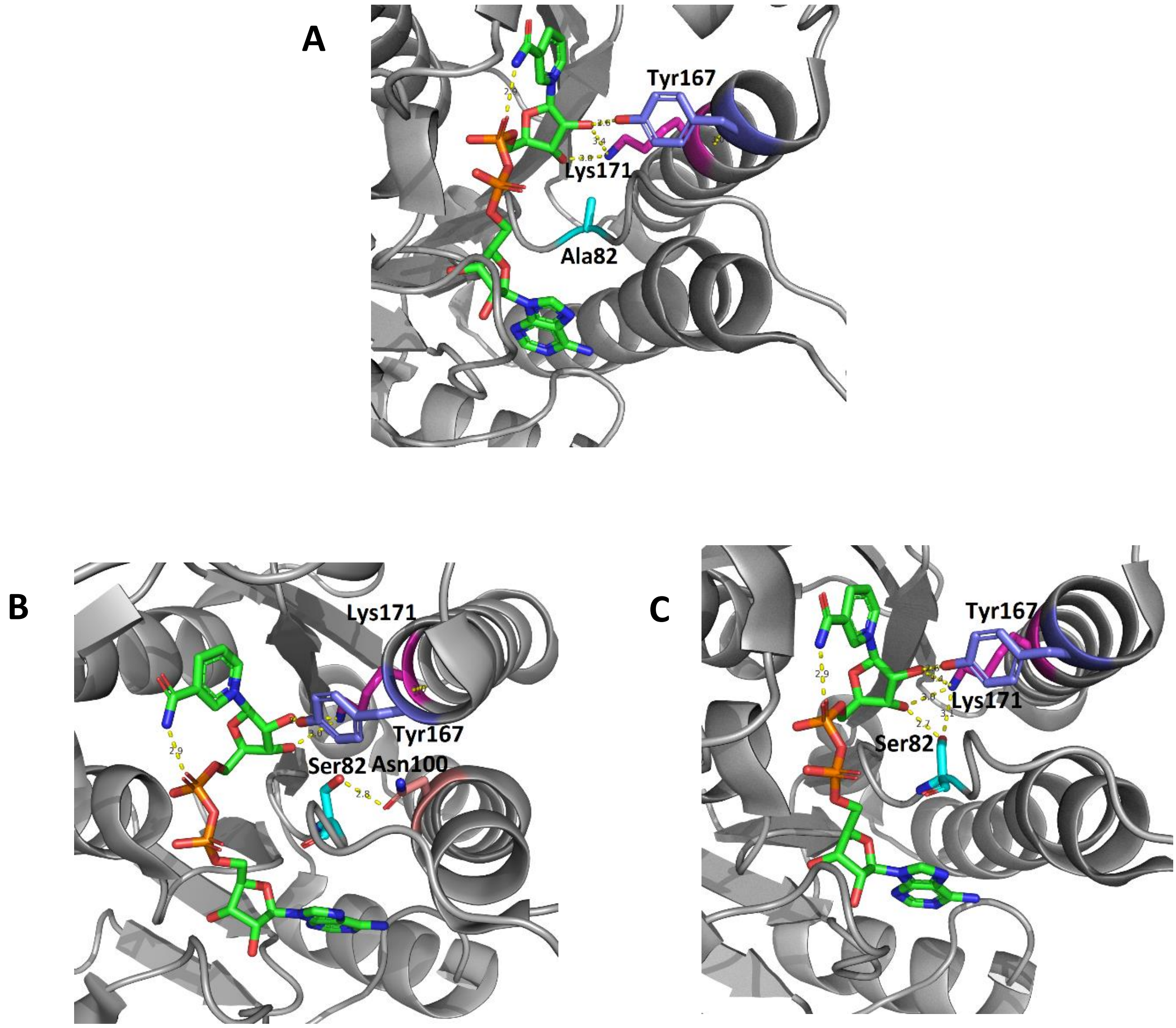

Figure S4. Close view of the coenzyme binding pocket of *S. enterica* RmlB and the predicted Ala82Ser mutant.
The residues Tyr167 and Lys171, belonging to the catalytic triad, interact with the 2' and 3'-hydroxyl groups of the nicotinamide ribose. (A) Wild type; (B) and (C) different models of the Ala82Ser mutant for two possible Ser82 rotamers obtained by *in silico* mutagenesis using PyMOL. *S. enterica* serovar Typhimurium: pdb 1G1A.

Supporting Figure S5

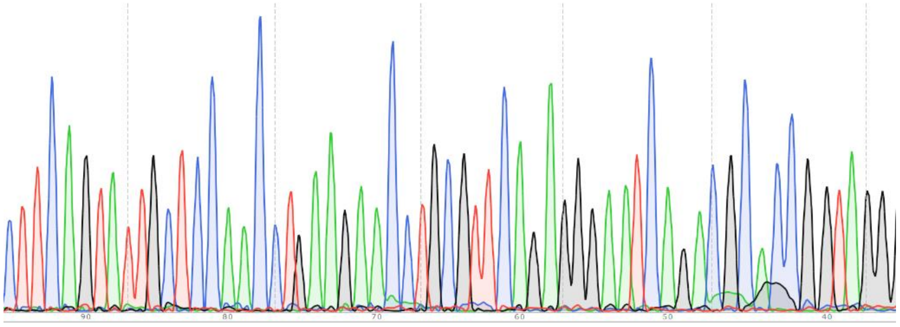

EXON 4 ..*CAGTATTGCTCCAACCTGAAGAACCTGCGTTCAGTCC***AGG***CCAGCAGCTCTTAC*..
DEL 4-12 ..CAGTATTGCTCCAACCTGAAGAACCTGCGTTCAG**AGG**GAATCAGACGCACCG..
EXON 12 ..CACAGACTCGGCTGGAG*ACCCAAAGTGGCCTGGACAG***AGG**GAATCAGACGCACCG..

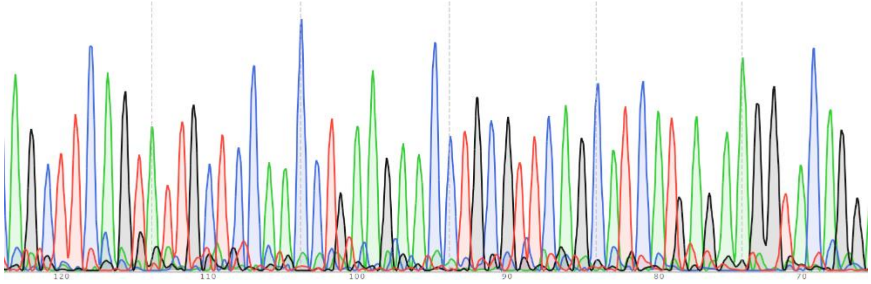

EXON 4 ..GTATTGCTCCAACCT*GAAGAACCTGCGTTCAGTCC***AGG**CCAGCAGCTCTTAC..
DEL 4-8 ..GTATTGCTCCAACCTGAAGAACCTGCGTTCAGCATCATGAGAAG..
EXON 8 ..CCGTGATCACGAGGA*GCAGTAATGTGTACGGGCCG***CGG**CAGCATCATGAGAAG..

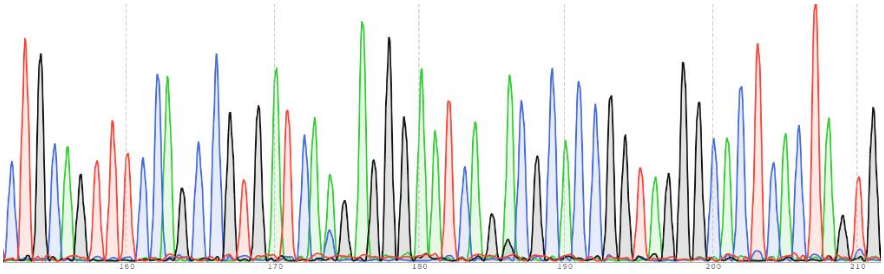

EXON 8 ..CCGTGATCACGAGGA*GCAGTAATGTGTACGGGCCG***CGG**CAGCATCATGAG..
DEL 8-12 ..CCGTGATCAAG**AGG**GAATCAGACGCA..
EXON 12 ..CAGACTCGGCTGGAG*ACCCAAAGTGGCCTGGACAG***AGG**GAATCAGACGCA..

Figure S5. Representative results obtained from Sanger sequencing of the PCR products derived from genotyping.
Representative PCR products of the deletions used for sequencing are reported in Figure 7. The gRNA target sequence is underlined, and the PAM is indicated in bold.

Supporting Figure S6

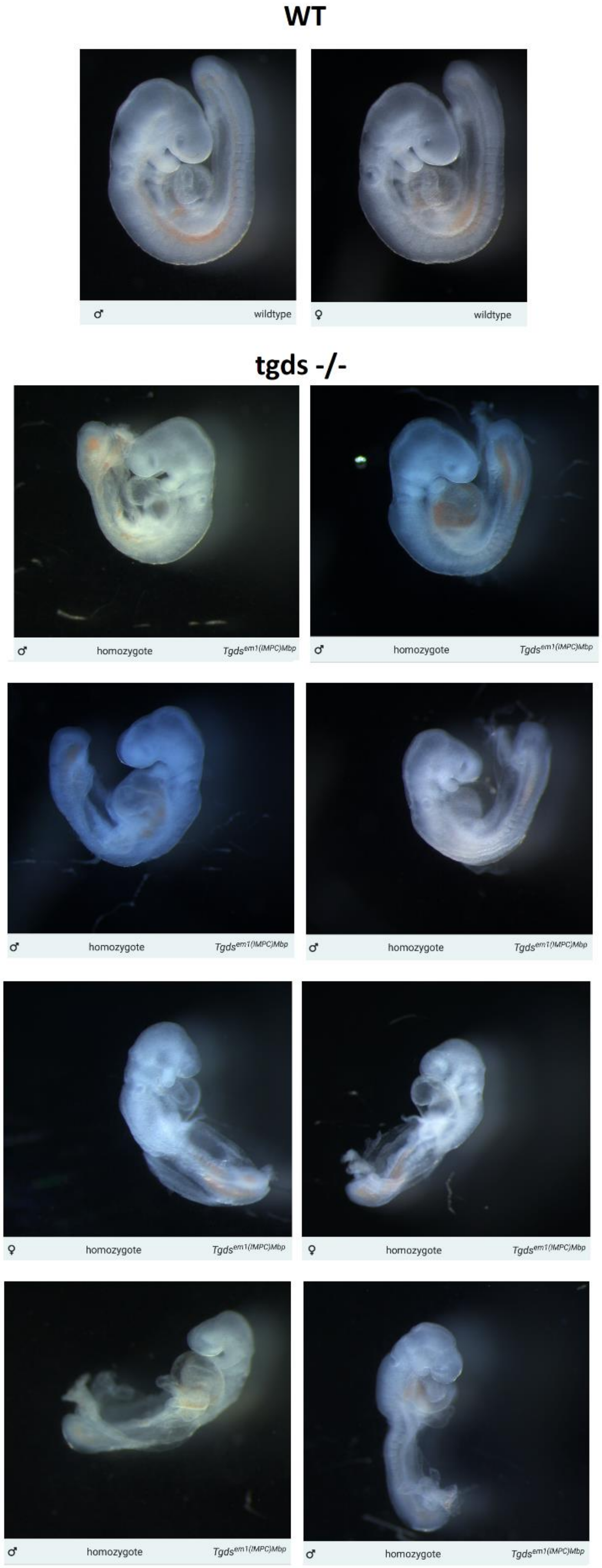

Figure S6. Representative images for WT and *tgds* knock-out mice obtained from International Mouse Phenotyping Consortium.

Images at E9.5 were obtained from IMPC (www.themousephenotype.org), under a Creative Commons Attribution 4.0 International license (CC-BY 4.0) [28].

Supporting Figure S7

120 hpf

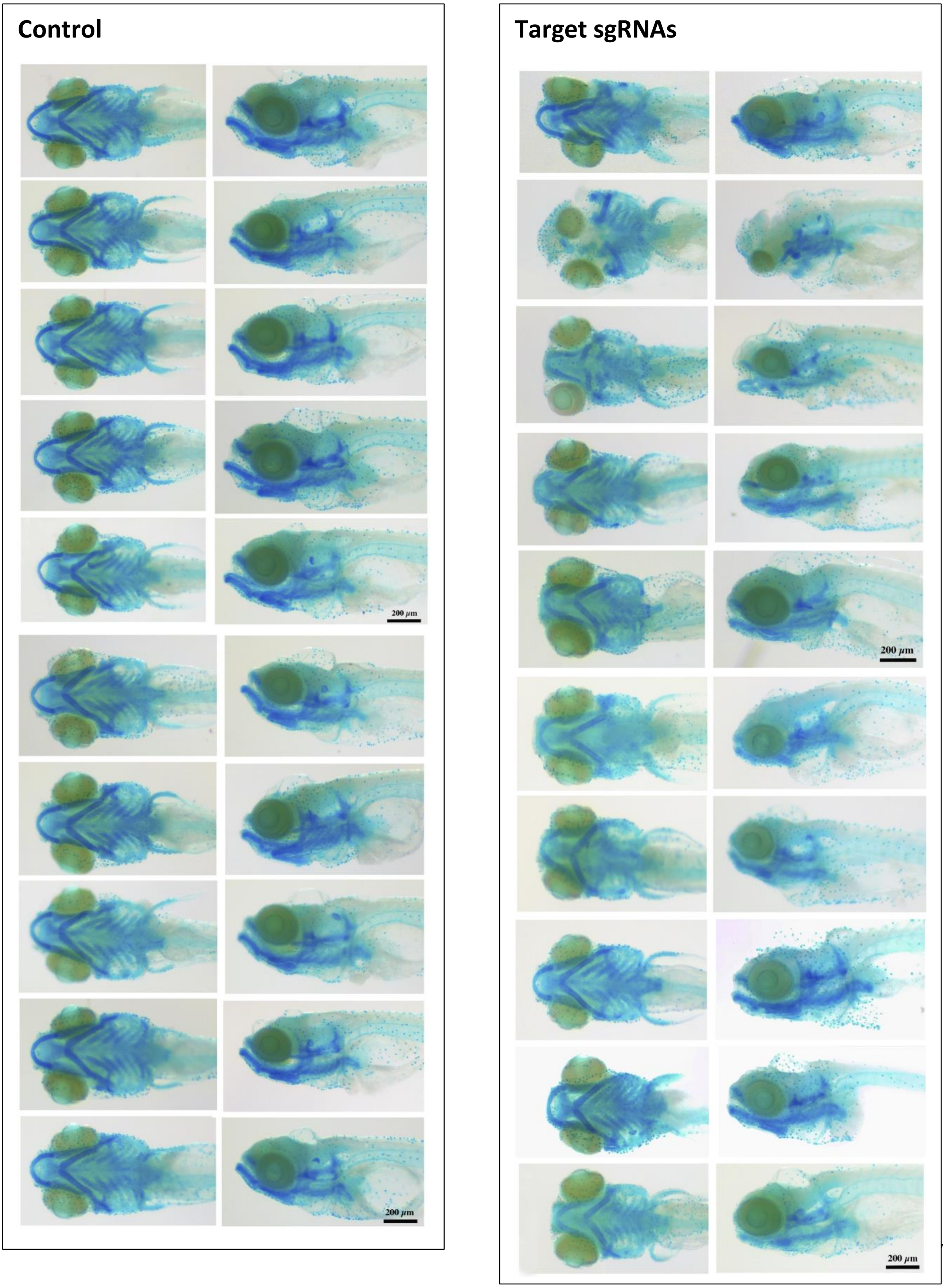

Supporting Figure S7 (continued)

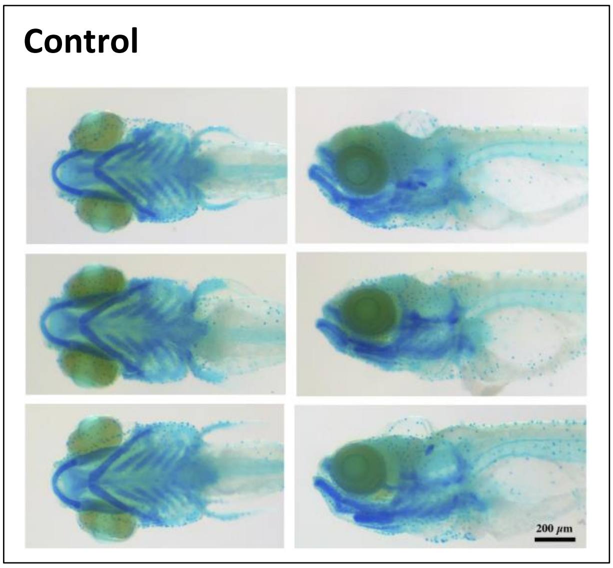

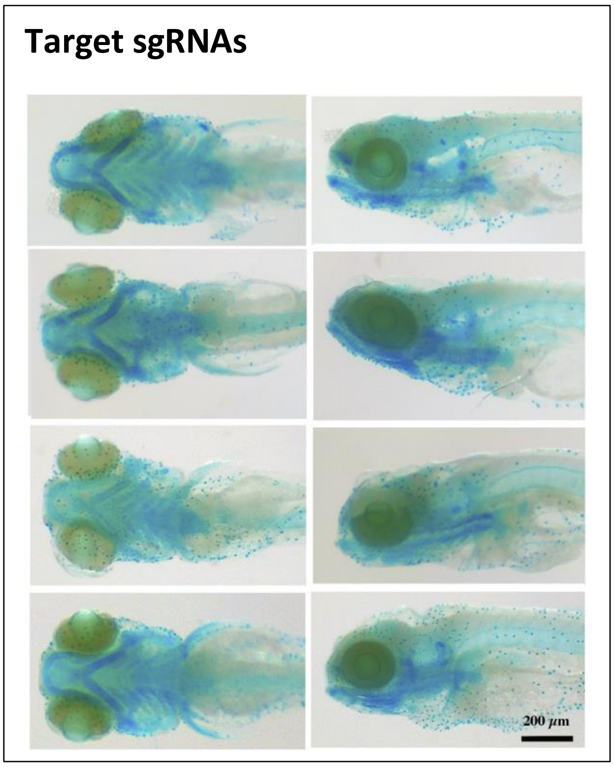

10 dpf

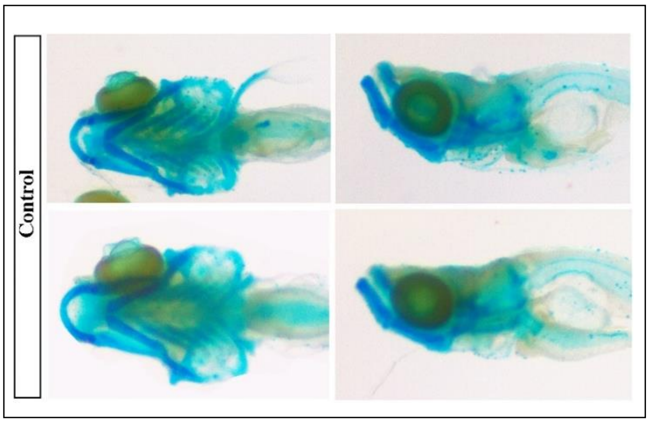

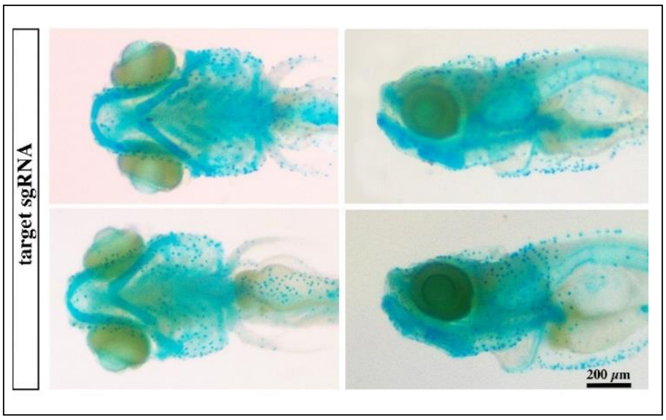

Figure S7. Alcian blue staining of further examples for controls and target sgRNA injected embryos
Embryos were stained at 120 hours post-fertilization (hpf) and 10 days post-fertilization (dpf).

Supporting Table S1. sgRNA and PCR primers for CRISPR-Cas9 and genotyping

gRNA sequence and CHOCHOP parameters

sgRNA	Sequence	Strand	%GC content	Predicted mismatches				Efficiency
				MM0	MM1	MM2	MM3	
Ex4	GAAGAACCTGCGTTCAGTCC	+	55	0	0	0	0	0.64
Ex8	GCAGTAATGTGTACGGGCCG	+	60	0	0	0	0	0.66
Ex12	GCAGTAATGTGTACGGGCCG	+	60	0	0	0	0	0.65

Primers for genotyping

PCR primers	Sequence
EX4_For	GGTTTCCTCACTGGAGGATC
EX4_Rev	TACATGAGTTTCAGCAGCGC
EX8_For	CCCTTTGATGAGCTGAGTCC
EX8_Rev	GCGAGAGGAATCTGGGAATG
EX12_For	GCTGAATCTCTGGACGACTG
EX12_Rev	TGTAACAGAGAGATTCCCTTCAG

Supporting Table S2. List of primers for RT-qPCR and cloning

Primers for RT-PCR and qRT-PCR

Gene name	Acc n°	Sequence (5′ → 3′)	Tm °C	Amplicon size (bp)
tgds	NM_001441693	TCCAAATGACAGTGAAGAGC	56	188
		TGGGTTCTCCTCGTACCACT	60	
beta actin 2 (actb2)	NM_181601	CCATCCTCCGTCTGGACTTG	60	301
		ATACCGCAAGATTCCATACCCAA	60	
eukaryotic translation elongation factor 1α1(eef1a1)	NM_131263	CTACCCTCCTCTTGGTCGCT	60	162
		GGAACGGTGTGATTGAGGGAA	60	

Primers for tgds cloning in pET28a

	Restriction site	Sequence (5′ → 3′)
Forward	NdeI	AATT**CATATG**AGCTGCAGTGACCGGCGGAC
Reverse	XhoI	AATT**CTCGAG**TTAGACTGGATGAGGCGTCTG

The restriction sites are indicated in bold

Primers for site directed mutagenesis

Mutant	Mismatch	Sequence (5′ → 3′)
Glu79Gly For	a236g_	AGACCACATCGATGTGTCCAGTGGAAAACAGATG
Glu79Gly Rev		GCATCTGTTTTCCACTGGACACATCGATGTGGTCT
Ala89Ser For	g265a_c266g_	TCTCTACATGAGTTTCACTAGCGCAGTGGAAGACCACATC
Ala89Ser Rev		GATGTGGTCTTCCACTGCGCTAGTGAAACTCATGTAGAGA
Tyr223His For	t667c_	GGTGACGTCAGAGACATGCAGGAAGTGTCTTGACT
Tyr223His Rev		AGTCAAGACACTTCCTGCATGTCTCTGACGTCACC
